# Recent advances in xylochemicals of interest to the paints and coatings industry

**DOI:** 10.1039/d6gc02642g

**Published:** 2026-08-17

**Authors:** Miłosz J. Długosz, Jake M. Graham, Megan S. Smith, Harry Maslen, Helen F. Sneddon

**Affiliations:** a Chemistry Research Laboratory, Department of Chemistry, University of Oxford Oxford OX1 3TA UK; b Green Chemistry Centre of Excellence, Department of Chemistry, University of York Heslington York YO10 5DD UK helen.sneddon@york.ac.uk

## Abstract

Xylochemicals, bio-based platform molecules isolated from wood-based waste streams, have been of particular interest to those in the paints and coatings industry seeking to increase bio-based content in their products. This tutorial review provides an introduction to the area, and covers recent advances across three key platforms: furfural and 5-hydroxymethylfurfural, vanillin, and cardanol derivatives, as well as an overview of other lignocellulosic platform molecules currently under investigation.

## Abstract 2

Xylochemicals, bio-based platform molecules isolated from wood-based waste streams, especially furfural and 5-hydroxymethylfurfural, vanillin, and cardanol derivatives, have recently been of particular interest to the paints and coatings industry.

## Graphical abstract

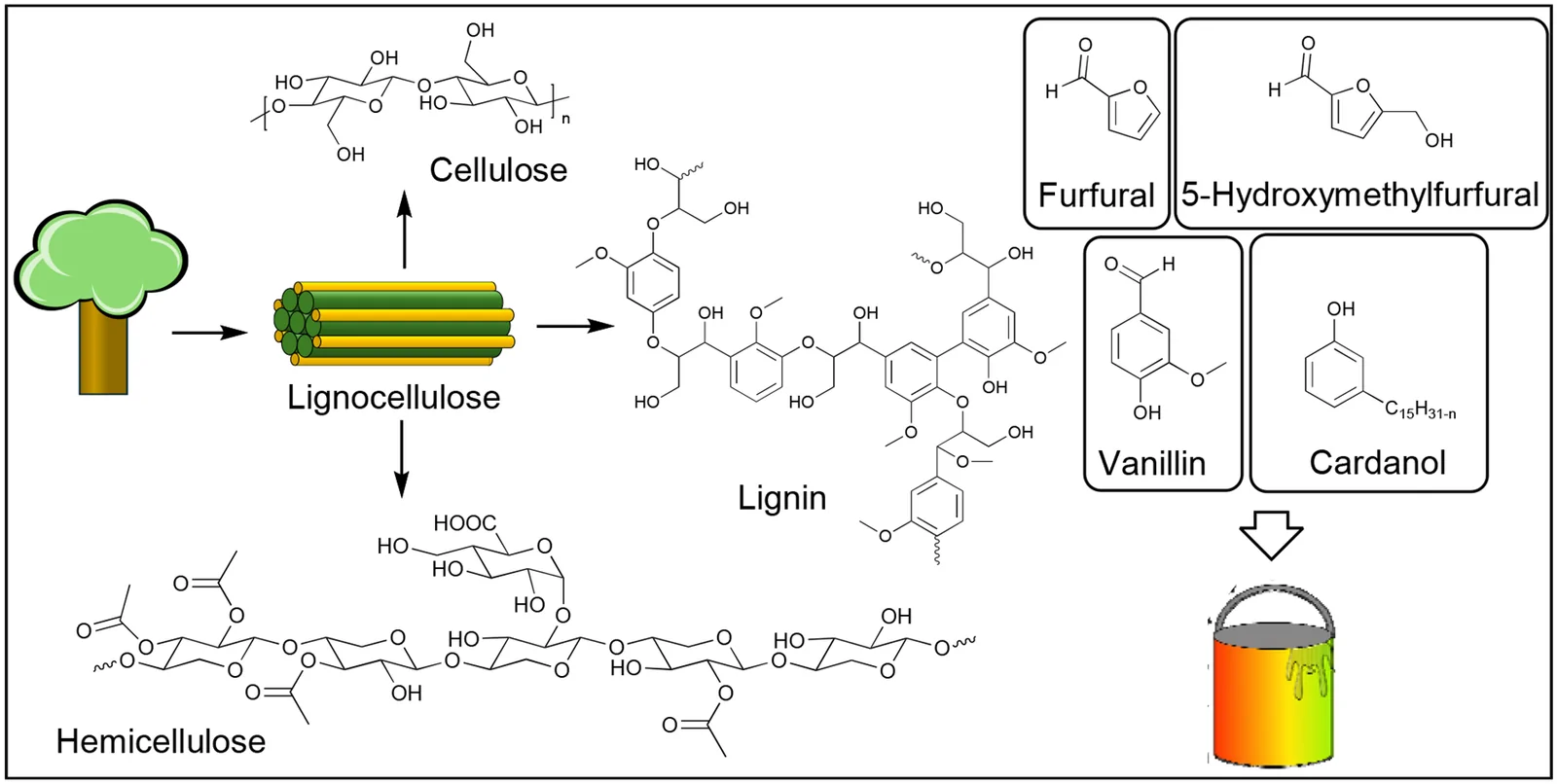

**Green foundation**

1. Recent advances in the incorporation of xylochemicals, (bio-based platform molecules isolated from wood-based waste streams) across three key platforms: furfural and 5-hydroxymethylfurfural, vanillin, and cardanol derivatives into the paints and coatings industry are discussed.

2. The high value and high tonnage of the paints and coatings industry makes this an important area for the increased use of bio-based, defossilised, platform molecules.

3. This review, focusing on 3 particularly promising platforms, emphasizes the diversity of ways in which a handful of structures can be applied to paints and coatings applications, and calls on industry and academia (ideally with incentives) to demonstrate the viability of these and similar approaches in order that their application on scale might meet the challenge of being competitive with large scale, long-established, petrochemical alternatives.

## 1. Introduction

The chemical industry has long relied on fossil-derived carbon as the feedstock for the majority of synthetic routes. Approximately 9% of the fossil fuels extracted each year are used to produce chemicals, accounting for an annual production of 330 million tonnes of petrochemicals.^1^ Through reforming and cracking, petroleum is converted into seven simple molecules known as base chemicals: methanol, ethene, propene, butadiene, benzene, toluene, and xylene. These simple base chemicals are converted to the incredible range of molecules responsible for modern society *via* chemical transformations.^2^

Although the chemical industry is currently largely dependent upon fossil fuels to meet our needs, this is not a sustainable solution. It is well established that fossil fuels are a finite resource, with the concept of “peak oil” being discussed as early as 1956,^3^ and current geopolitical tensions in the Strait of Hormuz increasing public awareness of the degree to which much of modern society (energy transport and base chemicals) is built on oil and gas.

The desire to move away from petroleum for our chemical needs has led to a focus on sustainable alternatives with the aim of achieving a circular economy. The Twelve Principles of Green Chemistry were established by Anastas and Warner in 1998, acting as guidelines for the development of more environmentally-friendly chemical syntheses and technologies.^4^ Principle 7 focuses on the use of renewable feedstocks as a source of raw materials for syntheses and chemical transformations. The most common approach to achieving this principle is to use biomass as a feedstock.^5^ The structures of molecules extracted from biomass are typically more complex than petroleum-derived base chemicals, often containing higher heteroatom content.^6^ This allows for the extraction of highly functionalised bio-based building blocks, known as platform molecules, that can be used as starting points for chemical syntheses.

Wood waste streams, for example, exist as a byproduct of paper production and horticulture, and the chemicals that can be extracted from wood feedstocks are known as xylochemicals.^7^ Such feedstocks can therefore be used to simultaneously minimise waste and supplement the chemical industry. Unlike crop-based feedstocks, the use of wood waste streams ensures there is no inherent competition with food production thereby side-stepping the ethical debate of “food *versus* fuel”.

**Fig. 1:**
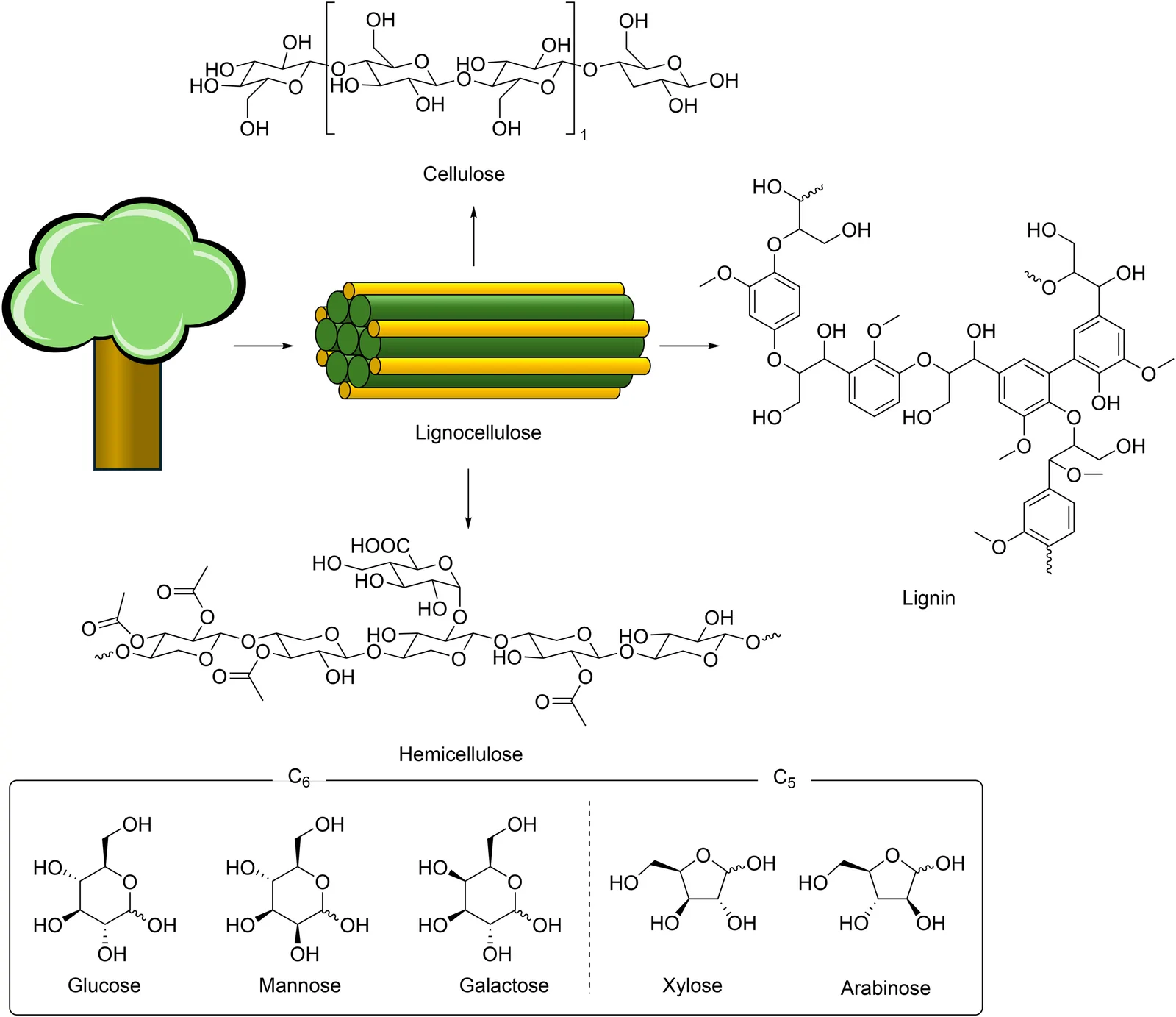
General structure and components of lignocellulose and its components (chemical structures redrawn, based on Iskigor and Becer).^14^

Wood has a high lignocellulose content, the three components of which are: cellulose, a polymer of glucose; hemicellulose, a series of branched polysaccharides; and lignin, a highly complex crosslinked aromatic polymer (Fig. 1). Cellulose and hemicellulose serve as sources of many sugars, providing highly functionalised structures in relatively few synthetic steps. Lignin, on the other hand, acts as a natural source of aromatic compounds. It should be noted however, that woody biomass is not the only source of xylochemicals, with chemicals such as cardanol also extracted from other bio-derived sources, such as cashew nut shell liquid.^8^ Xylochemicals can be categorised as either primary, extracted directly from biomass, or secondary, synthesised *via* simple transformations from the primary xylochemical. The reader is referred to a comprehensive review on xylochemicals by Arduengo and Opatz, which provides over 100 examples of xylochemical structures and their sources.^7^

Protective coatings are applied to surfaces to act as a physical barrier against damage. By preventing erosion, corrosion and other forms of damage, a coating can increase the lifespan of a material, avoiding or delaying the need to replace or repair a structure. Coatings can also confer other physical properties onto a structure, such as hydrophobicity and electrical insulation.^9^

In the context of green chemistry, the paints and coatings industry is a field that has been given much discussion.^10,11^ Whilst not all paints and coatings use solvents, due to the size of the sector and the application of coatings often requiring high volumes of solvent, paints and coatings account for 57% of the global market share of solvents.^12^ Due to their significant volume, efforts have been made to improve the sustainability of the solvents used in paints and coatings.^13^

Although solvents comprise the bulk of a coating formulation by volume, the sustainability of the other components is also critical. The second (or third after pigment) largest component of a coating is the polymeric resin, responsible for the basic chemical and physical properties of the coating.^15^ A range of polymeric architectures can be used as resins, such as epoxy or polyester resins, commonly used for anti-corrosive coatings on metal, or polyurethane (PU) coatings, widely used in the automobile industry.^16–18^

A key component of some coatings is a cross-linker or hardener, a low molecular weight polymer with functional groups capable of fusing larger polymer chains. They react with the resin to create a sturdier polymer network providing mechanical and solvent resistance to the coating.^19^

Both resins and cross-linkers are currently predominantly made from petrochemicals, and the library of xylochemicals on the market offers potential for the synthesis of bio-based resins and cross-linkers for paints and coatings. Many existing resins contain aromatic structures, and this aromatic component has been correlated with mechanical hardness. Hence, aromatic structures present in lignin, or furfural derivatives extracted from hemicellulose, show potential as starting points for the replacement of petrochemical-derived resins.

This review aims to give an overview of recent developments in the field of xylochemicals, specifically those relevant to the paints and coatings industry. The sustainability of coatings is inherently multi-faceted – including solvent usage, the conditions and reagents required for polymer synthesis, and carbon footprint, and downstream factors including the duration of the coating, sustainability benefits incurred, biodegradability, toxicity *etc*. – this review focuses primarily on the applications of three specific bio-based chemicals in this space. A more in-depth study and life cycle assessment beyond the scope of this review would be required for each bio-based coating to determine if it truly is “sustainable”. Focussing on furfural-, vanillin- and cardanol-based resins due to their frequent appearances in recent literature, it is hoped that this review will offer an insight into existing strategies for the development of sustainable coatings. These chemicals are industrially relevant due to their current (or anticipated) large-scale production, making them among the more economically viable alternatives to petrochemical resins and likely targets for formulation in the next wave of sustainable commercial paints and coatings.

## 2. Furfural- and 5-hydroxymethylfurfural-derivatives

**Fig. 2:**
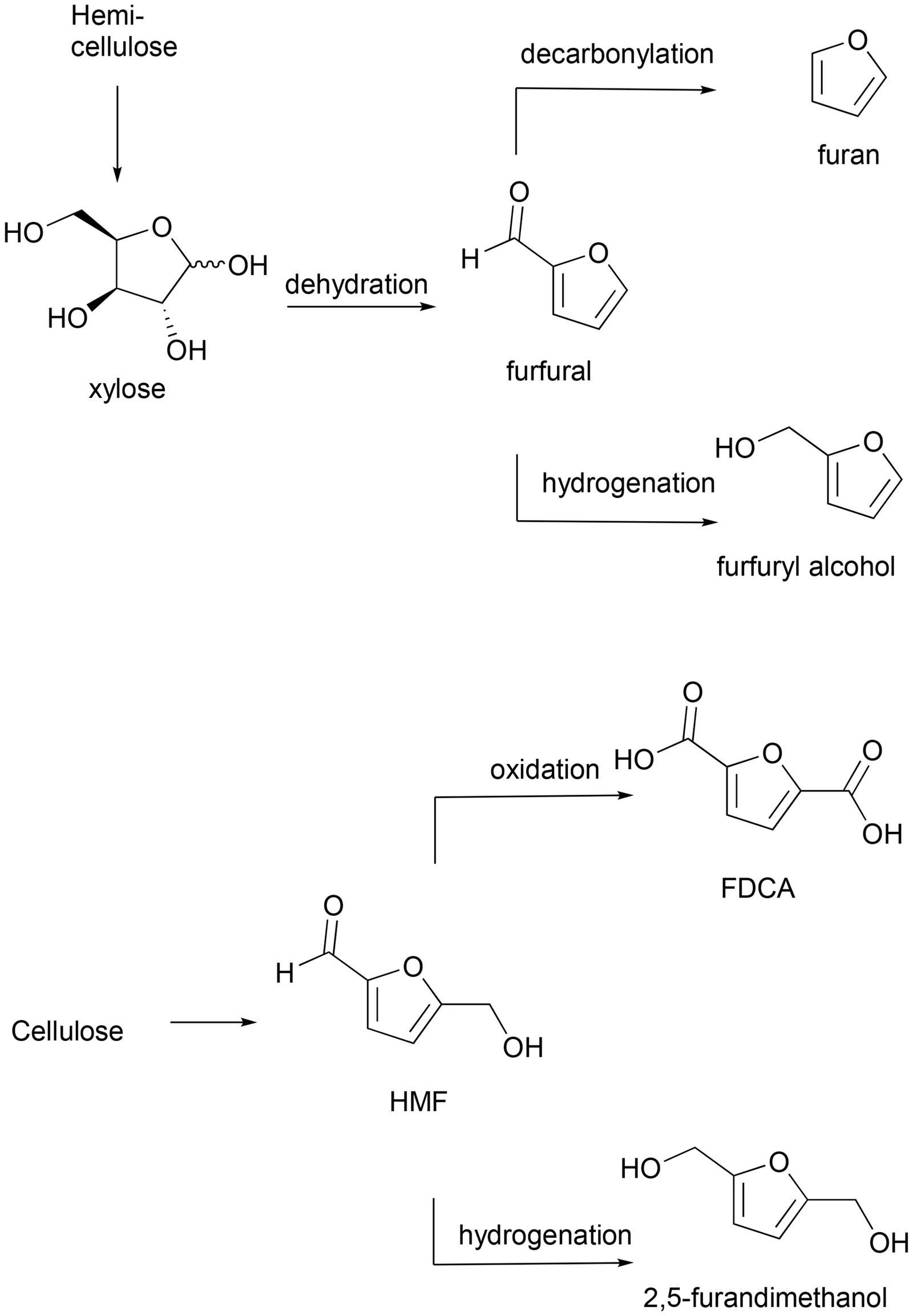
Extraction of furfural, HMF and derivatives from lignocellulosic biomass.

Furfural is derived from hemicellulose commercially, *via* the hydrolysis of pentosans, then dehydration of pentose sugars, *e.g.* xylose (Fig. 2).^20^

Due to the complex nature of hemicellulose, the extraction gives low yields and selectivity, and leads to the production of acidic wastewater.^21,22^ Furfural is a platform molecule for a range of valuable bio-derived molecules, such as furan (*via* decarbonylation) or furfuryl alcohol (*via* hydrogenation). In 2004, the National Renewable Energy Laboratory ranked furfural and 2,5-furandicarboxylic acid (FDCA) as two of the thirty most marketable and valuable building blocks from lignocellulosic biomass.^23^ As of 2021, the global market value of furfural reached $520 million, with an expected market value of $818 million by 2031.^24^ This accounts for approximately 300 000 tonnes of furfural produced per annum from biomass.^25^ Although 70% is produced in China, the Dominican Republic is home to the largest furfural production plant, accounting for 35 000 tonnes per annum.^26^

5-Hydroxymethylfurfural (HMF) is derived from the dehydrocyclisation of cellulose, one of the major components of lignocellulose.^27^ Although HMF itself has not found many direct applications, it can be converted to a range of desirable compounds, such as FDCA, which has found significant use in the polymer industry as a potential replacement for terephthalic acid.^28^ FDCA's high price††Prices quoted online on 27th April included £205.00 for 25 g (2,5-furandicarboxylic acid | 3238-40-2 | Tokyo Chemical Industry UK Ltd) and £188.00 for 5 g (2,5-furandicarboxylic acid 97 3238-40-2). can be attributed partially to a lack of industrial production. After demonstrating viability on a pilot scale, Avantium began development of the first industrial plant for the production of FDCA, aiming to produce 5000 tonnes per annum and commence sales in early 2026.^29,30^

**Fig. 3:**
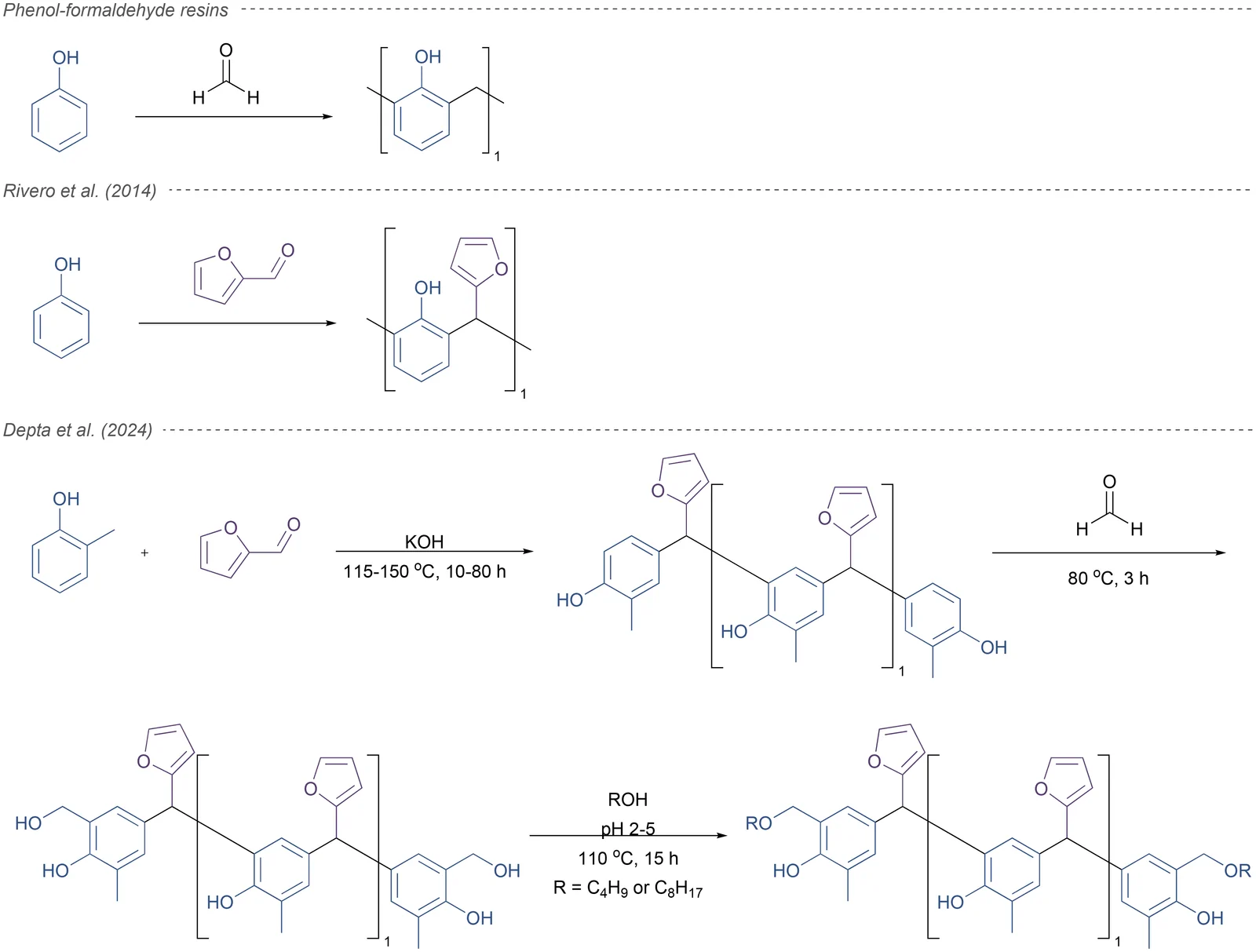
Phenol-formaldehyde, phenol-furfural resins and *o*-cresol-furfural-formaldehyde etherified resins.

In the paints and coatings industry, furfural derivatives have found many applications in the form of resins.^31^ Whilst the differences between heterocycles and aromatic rings are well documented, furans can be considered in some ways analogous to phenols in resin applications, as they both confer similar thermal and mechanical properties to polymeric materials due to their rigid aromatic structure.^32^ First discovered by Baeyer in 1872,^33^ phenolic resins have been produced on an industrial scale since the early 20^th^ century, famously including Bakelite, the world's first fully synthetic plastic material.^33^ Phenol-formaldehyde resins are synthesised by the simple condensation reaction between phenol and formaldehyde (Fig. 3) and have found applications in various protective coatings due to their anti-corrosive properties.^34^ However, the carcinogenicity of formaldehyde has led to strict EU regulations preventing the marketing of resins containing >0.1% unreacted formaldehyde, creating a gap for potential sustainable alternatives to replace formaldehyde in these resins.^35^

One potential formaldehyde replacement is furfural. Rivero *et al.* developed phenol-furfural resins (Fig. 3)^32^ as a more sustainable alternative to traditional phenolic coatings for aluminium.^32^ Displaying similar thermomechanical properties due to their similar structures, it was found that the phenol-furfural resin showed an improved hydrophilic character, attributed to the greater presence of polar groups. Both coatings provided similar degrees of mechanical protection when applied to aluminium, with the furan coating exhibiting high resistance to corrosion. This was further improved through the incorporation of cellulose nanocrystals (CNCs) into the resin structure.^36^ The abundance of hydroxyl groups in CNCs encouraged hydrophilic interactions between the resin and nanocrystals. This simple modification led to excellent adhesion to the aluminium surface with up to 20% CNC content, as well as improving resistance to mechanical damage, and maintaining thermal resistance to degradation.

Recent research into phenol-formaldehyde resins has focused on improving their properties *via* chemical modification, typically at the processing stage. One synthetic method of post-polymerisation modification that has been developed is the etherification of introduced hydroxymethylene groups, previously shown to improve the flexibility of varnish coatings.^37^ Aiming to adapt this to phenol-furfural resins, Depta *et al.* synthesised *o*-cresol-furfural resins.^35^*o*-Cresol is itself a xylochemical and can be derived from lignocellulose or wood pyrolysis, although commercial cresols are synthesised from petrochemical sources.^7^ After the synthesis, it was found that the resins did not contain any hydroxymethylene groups, and thus, etherification was not possible.^35^ In order to introduce hydroxymethylene sites for further modification, formaldehyde was added to the resin post-condensation (Fig. 3). Despite falling below the <0.1 wt% free formaldehyde limit, the use of formaldehyde would ideally be avoided entirely in any future work. The etherified resin was found to be more flexible and less viscous than the resin before modification, suggesting it could have potential uses as a protective coating for metal substrates.

**Fig. 4:**
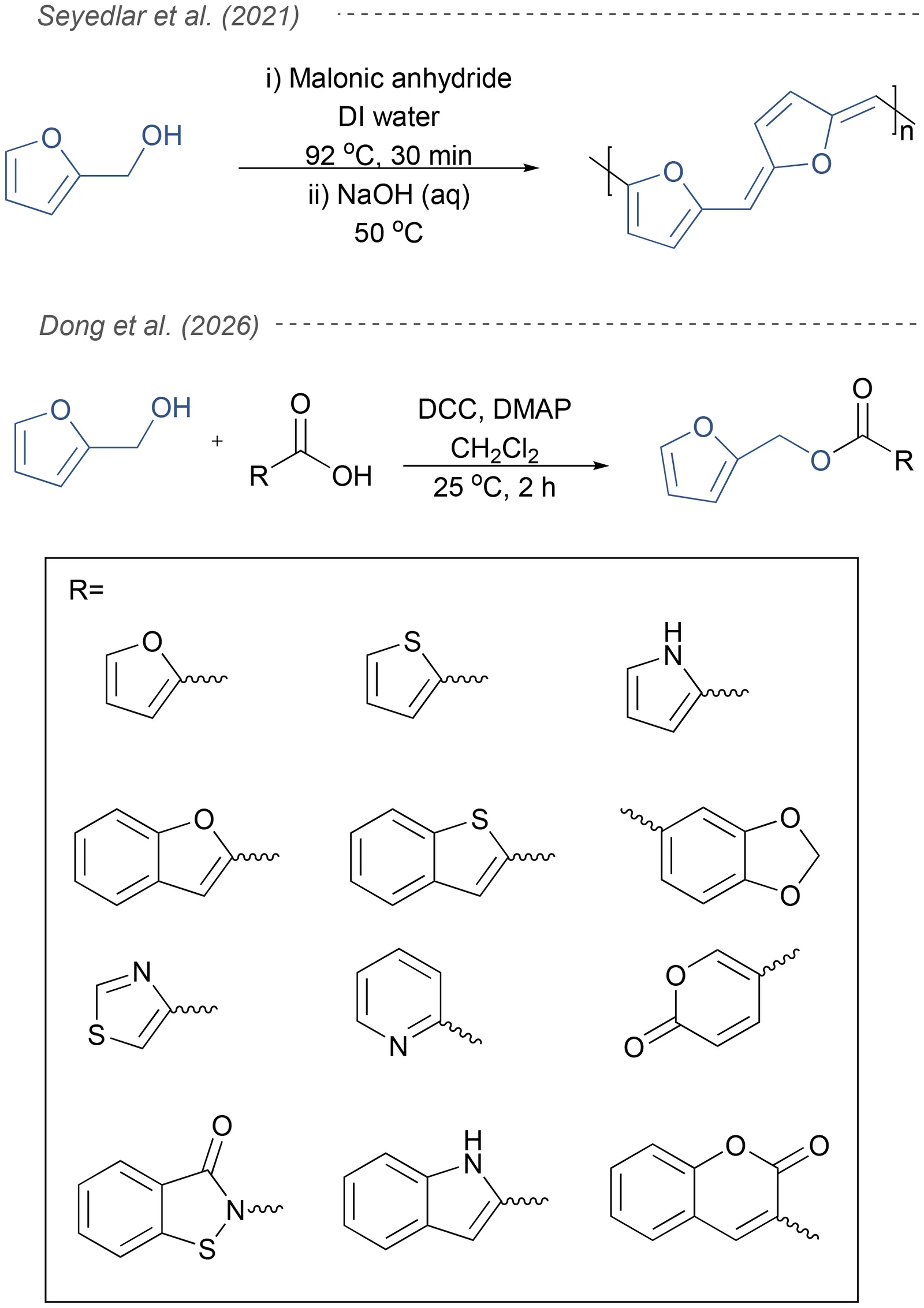
PFA resins and furfuryl alcohol-derived heteroaromatic molecules utilised as anti-fouling agents in marine coatings. DCC = dicyclohexylcarbodiimide. DMAP = 4-dimethylaminopyridine.

Poly(furfuryl alcohol) (PFA) resins have found commercial use in the polymer concrete industry due to thermal and environmental resistance, but have shown brittleness due to the rigid aromatic structure and cross-linking during polymerisation.^38,39^ This brittleness, as well as low viscosity, has prevented the use of PFA resins in coatings, as surface defects occur when applied. Seyedlar *et al.* investigated low molecular-weight PFA resins (Fig. 4) as an alternative to commercially available heat-resistant coatings, aiming to adapt the mechanical properties towards coating applications.^39^ Despite high thermal resistance, the PFA resin was too brittle and showed poor adhesion to the carbon-steel substrate. The addition of long-oil alkyd resin reduced cross-linking and acted as a plasticiser, increasing adhesion and flexibility.

Furfuryl alcohol has also been incorporated into a series of novel anti-fouling coatings.^40^ Natural products are often used as sustainable anti-fouling agents due to their degradability and biocompatibility,^41^ but issues with extraction and low abundance make them economically challenging to use on large-scale.^42^ Bio-based platform chemicals that contain similar structures could instead be employed to develop novel anti-fouling agents at lower costs.^43^ A series of 12 heterocyclic carboxylic acids were esterified with furfuryl alcohol (Fig. 4), and the products were combined with a zinc methacrylate resin, pigments, and dispersing agents to form anti-fouling coatings.^40^ The anti-fouling nature of these coatings is attributed to their inherent hydrophobicity, as well as the furan ring disrupting biofilm formation and preventing larvae from attaching to the surface.^44^ The heterocycles offer a diversity of structures that could also disrupt the fouling organisms’ metabolisms.^45^ The coatings exhibited high antimicrobial activity and demonstrated significantly reduced biofouling adhesion when compared to a control group. Future work aims to extend this approach to freshwater coatings.

**Fig. 5:**
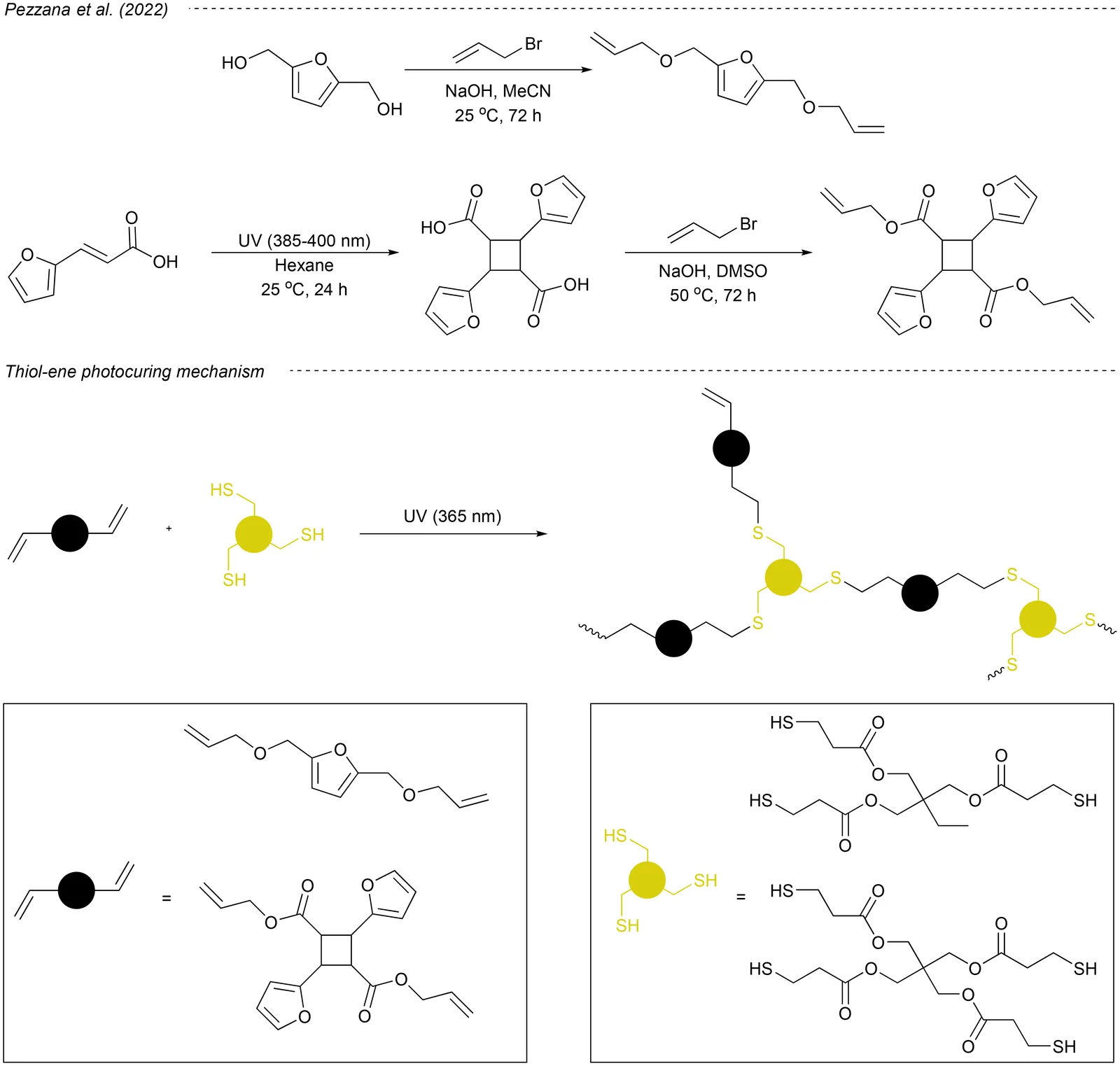
Synthesis of allylated monomers derived from FDM and FAA and the mechanism of thiol–ene UV-catalysed photocuring. DMSO = dimethyl sulfoxide.

UV-curable resins derived from HMF and furfural were synthesised by Pezzana *et al.*^46^ Two monomers were synthesised, derived from 2,5-furandimethanol (FDM) and 2-furanacrylic acid (FAA), respectively, and were cross-linked with thiol monomers through UV-catalysed thiol–ene click chemistry to form a photocured coating (Fig. 5). The more rigid structure of the FAA-derived monomer led to better mechanical properties over the FDM-derived monomer, and outperformed existing UV-curable coatings.

**Fig. 6:**
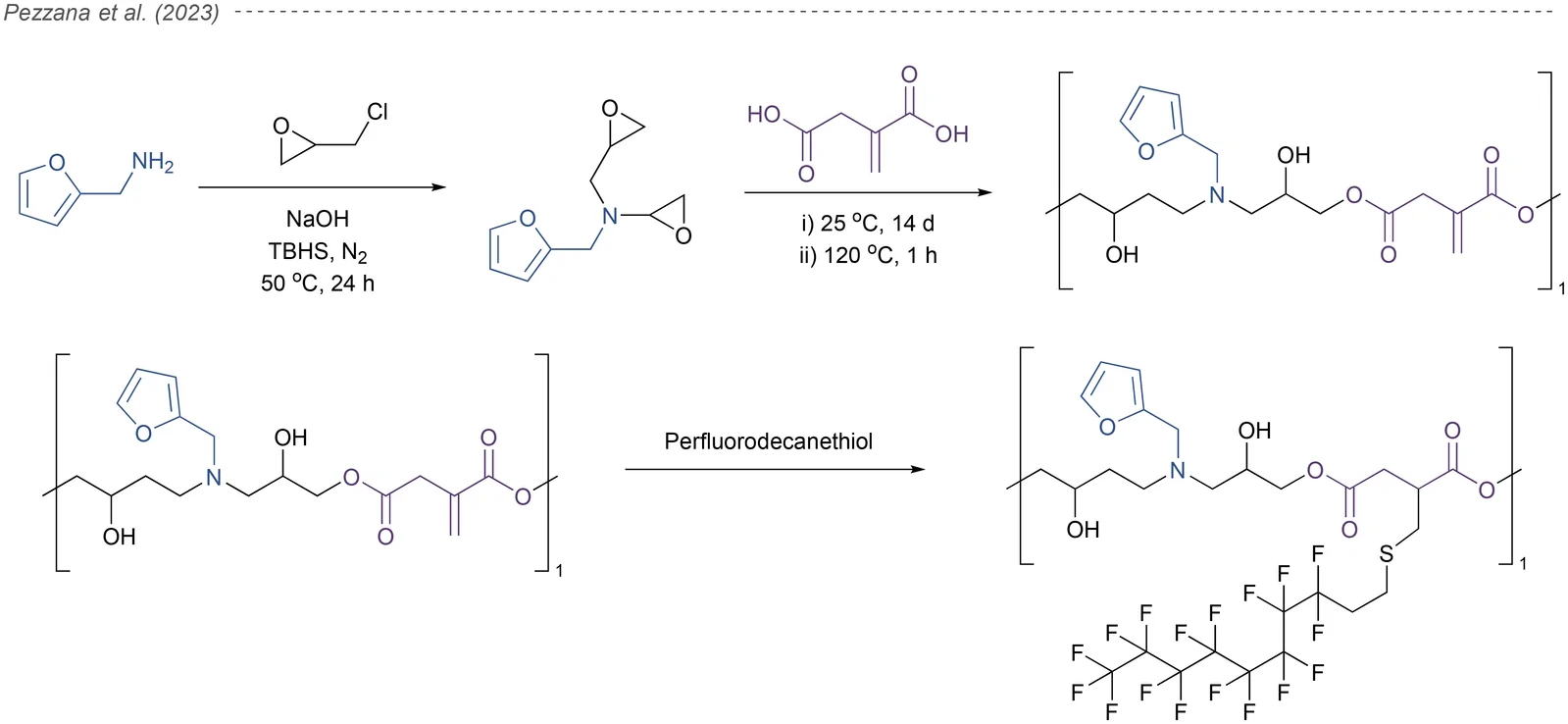
Furfurylamine and itaconic acid-derived resins with and post-polymerisation modification. TBHS = tetrabutylammonium hydrogen sulfate.

Thiol–ene reactivity has also been employed on resins derived from furfurylamine to produce functional coatings.^47^ Furfurylamine is typically synthesised from the reductive amination of furfural with ammonia, but a common issue with this reaction is over-reduction and loss of aromaticity.^48^ Recent developments have shown that ω-transaminase enzymes can be utilised to avoid these harsh conditions, although typically an excess of the amine donor is required to achieve high yields.^49^ Pezzana *et al.* synthesised a diglycidyl furfurylamine monomer, which was thermally cured with itaconic acid (derived from the fermentation of glucose)^7^ at room temperature.^47^ The presence of pendant unsaturated double bonds along the polymer backbone allowed for post-polymerisation functionalisation *via* thiol–ene chemistry. This strategy was utilised to graft perfluorodecanethiol onto the coating, altering the nature of the surface from hydrophilic to hydrophobic (Fig. 6). The success of the post-polymerisation functionalisation shows the potential this strategy offers for altering the properties of resins for a range of applications.

**Fig. 7:**
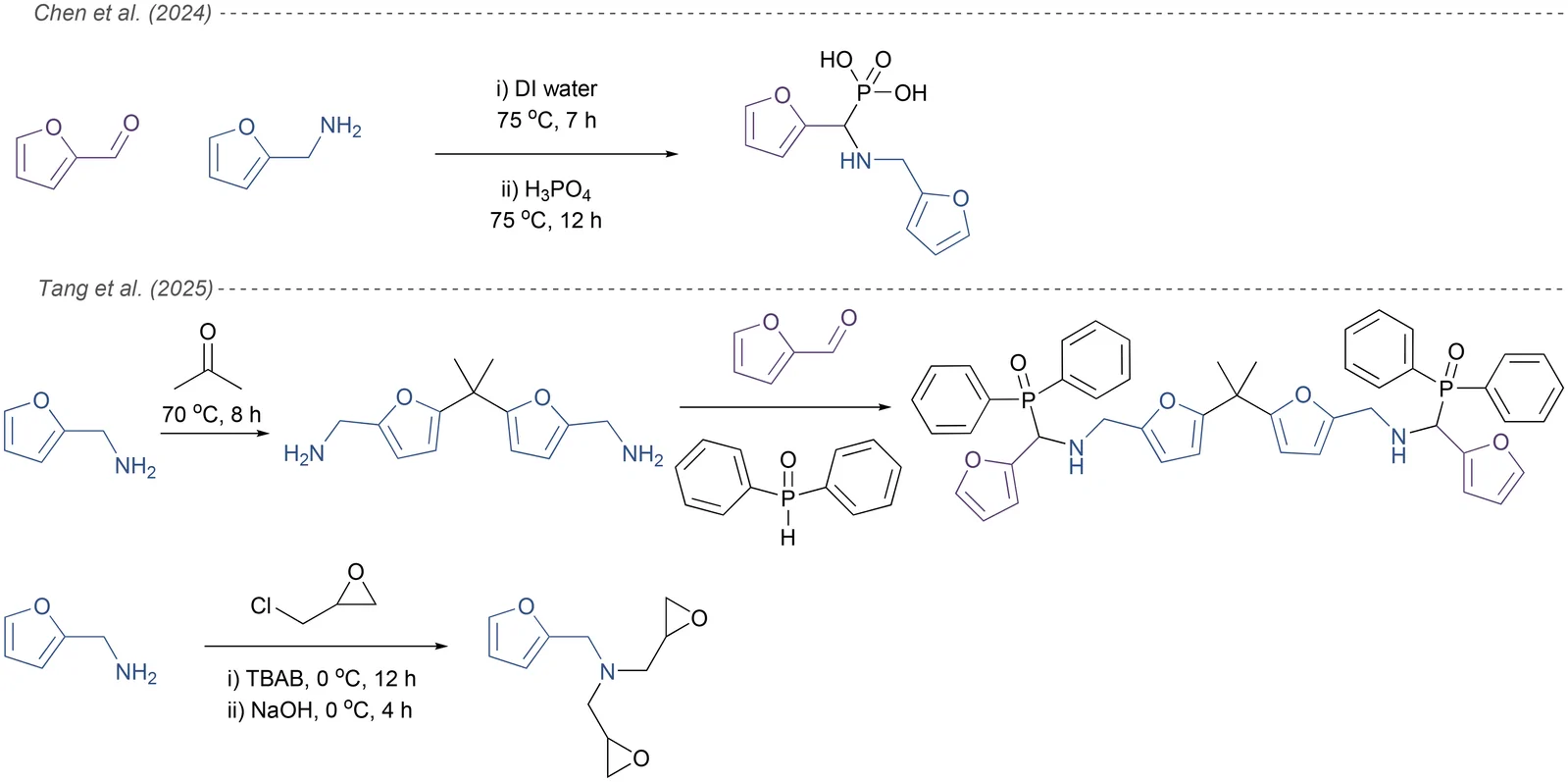
Furfurylamine- and furfural-derived phosphorylated monomers and furfurylamine epoxy resins for flame-retardant coatings. TBAB = tetrabutylammonium bromide.

Chen *et al.* developed a furfurylamine phosphorylated flame-retardant coating for cotton and poly(lactic acid) (PLA) fabrics.^50^ Testing of the flame-retardant properties showed a 69.4% and 37.8% reduction in the peak heat release rate of coated cotton and PLA fabrics, respectively (Fig. 7).

Epoxy resins are used in a variety of applications due to their mechanical properties and thermal and corrosion resistance. Approximately 90% of epoxy resins are bisphenol A diglycidyl ether (DGEBA) resins. Intending to replace bisphenol A (BPA)-derived resins, Tang *et al.* developed a furfurylamine-derived phosphine epoxy resin.^51^ The structure of the furfurylamine-derived phosphine epoxy resin was designed with flame-retardant properties in mind (Fig. 7).

**Fig. 8:**
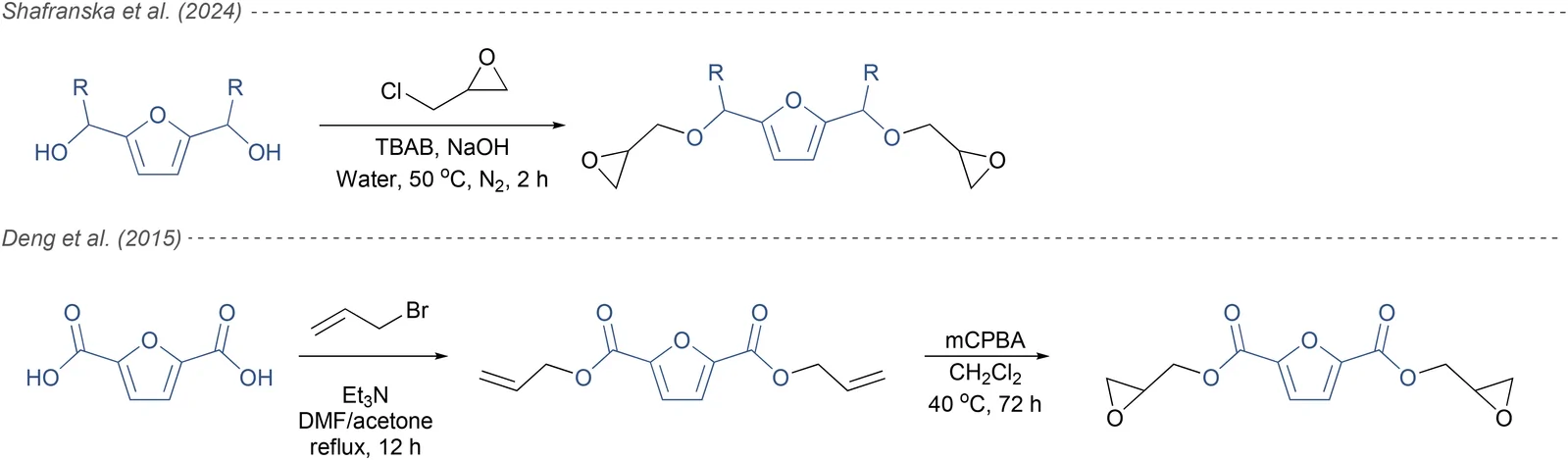
Epoxy resins derived from HMF and FDCA. TBAB = tetrabutylammonium bromide. DMF = dimethylformamide.

The reaction of HMF or 2,5-diformylfuran (accessed *via* the oxidation of HMF) with Grignard reagents has been used to form a range of asymmetric and symmetric diols, respectively.^52^ These furanic diols have been utilised to form epoxy resins for thermoset coatings by Shafranska *et al.* (Fig. 8).^53^ The diols were reacted with epichlorohydrin to form diglycidyl ethers before subsequent polymerisation with isophorone diamine to produce an epoxy resin. Applied to a steel surface, the cured thermosets demonstrated good adhesion, hardness, thermal stability and impact resistance. Due to the expected reduced toxicity compared to BPA, it was hoped that these resins could find applications where food and drink contact may occur, such as in tinned goods.

Additionally, FDCA has found applications in the coatings industry. Early work involved the diglycidyl ester of FDCA (DGF), which was synthesised on a multi-gram scale by Deng *et al.* and evaluated as a replacement for the petrochemical-derived diglycidyl ester of terephthalic acid (DGT) for coating applications (Fig. 8).^54^ The bio-based DGF exhibited a higher curing reactivity and *T*_g_, with both resins showing similar mechanical properties, and thermal stability. Despite the promising properties of the resin, it is worth noting the use of non-preferred solvents *N*,*N*-dimethylformamide and dichloromethane in the synthesis of the resin.

**Fig. 9:**
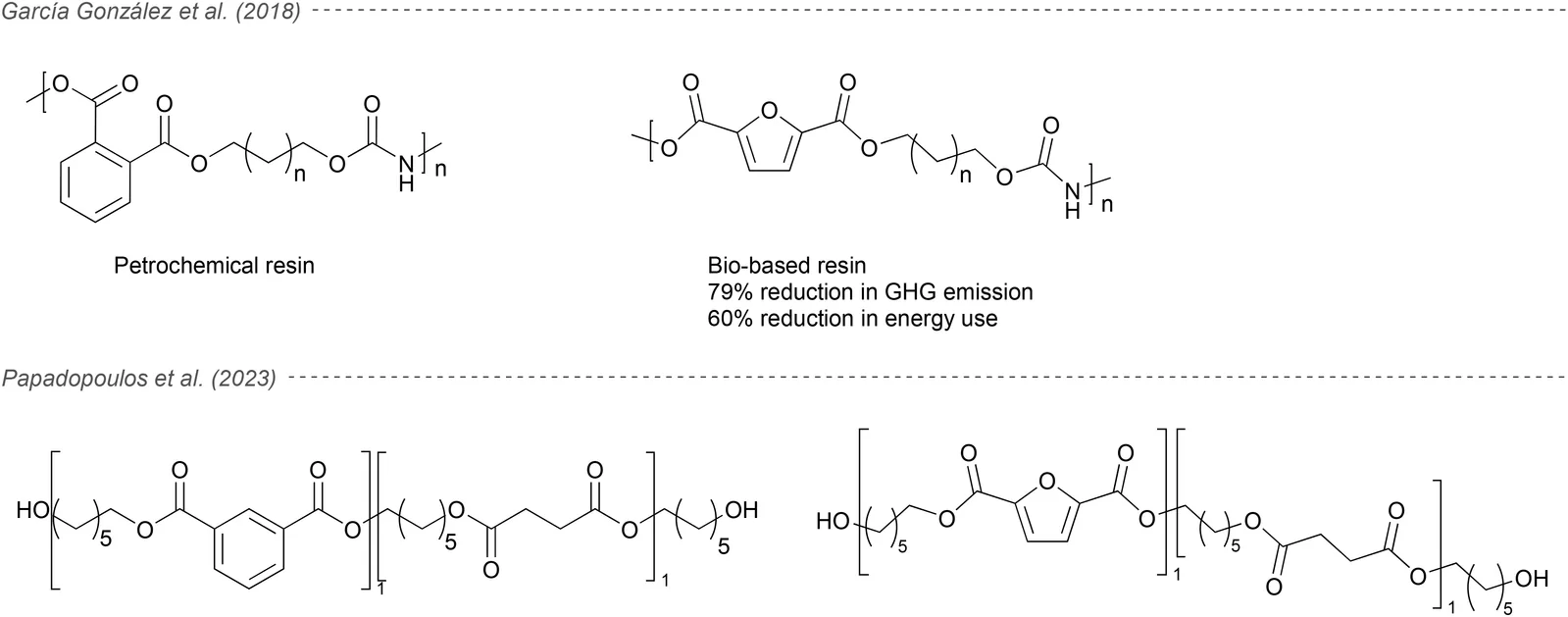
Polyurethane coatings derived from phthalic acid and FDCA, and WPU dispersions derived from terephthalic acid and FDCA.

Polyurethanes (PU) are polymers that contain a carbamate functional group, mostly synthesised from the reaction of oligomeric esters or polyols. PU coatings have a strong history of use in the automotive and furniture industries. García González *et al.* developed and performed a life cycle assessment of a fully bio-based polyester precursor to PUs, derived from FDCA, succinic acid, glycerine and 1,3-propanediol.^55^ The polyester was crosslinked with an aliphatic poly(isocyanate). This structure was found to produce a more hydrophilic PU coating than a terephthalic acid-derived equivalent PU (Fig. 9), demonstrating better adhesion to the surface but more moisture sensitivity. The substitution of terephthalic acid for FDCA led to a 79% reduction in Greenhouse Gas (GHG) emissions and a 60% reduction in energy use.

Recent advancements have centred on water-borne polyurethane dispersions (WPUs) as a means of reducing organic solvent waste. Papadopoulos *et al.* developed WPUs derived from FDCA, succinic acid and 1,6-hexanediol (Fig. 9).^56^ In comparison with PUs containing isophthalic acid, the FDCA-based dispersion exhibited improved properties, including a higher *T*_g_ and tensile modulus.

FDCA has also been tested for use in the development of coil coatings. When compared with a terephthalic acid-derived resin, resins with up to 31% FDCA content could achieve similar properties to the petrochemical resin in all assessed properties except for discolouration. It was found that the low FDCA-content resins discoloured over time, however, it was proposed that this could be avoided by a reduction in synthesis temperature and by the removal of terephthalic acid from the resin structure. Such coil coatings may have potential uses in the automotive or construction industries with further advancement.^57^

**Fig. 10:**
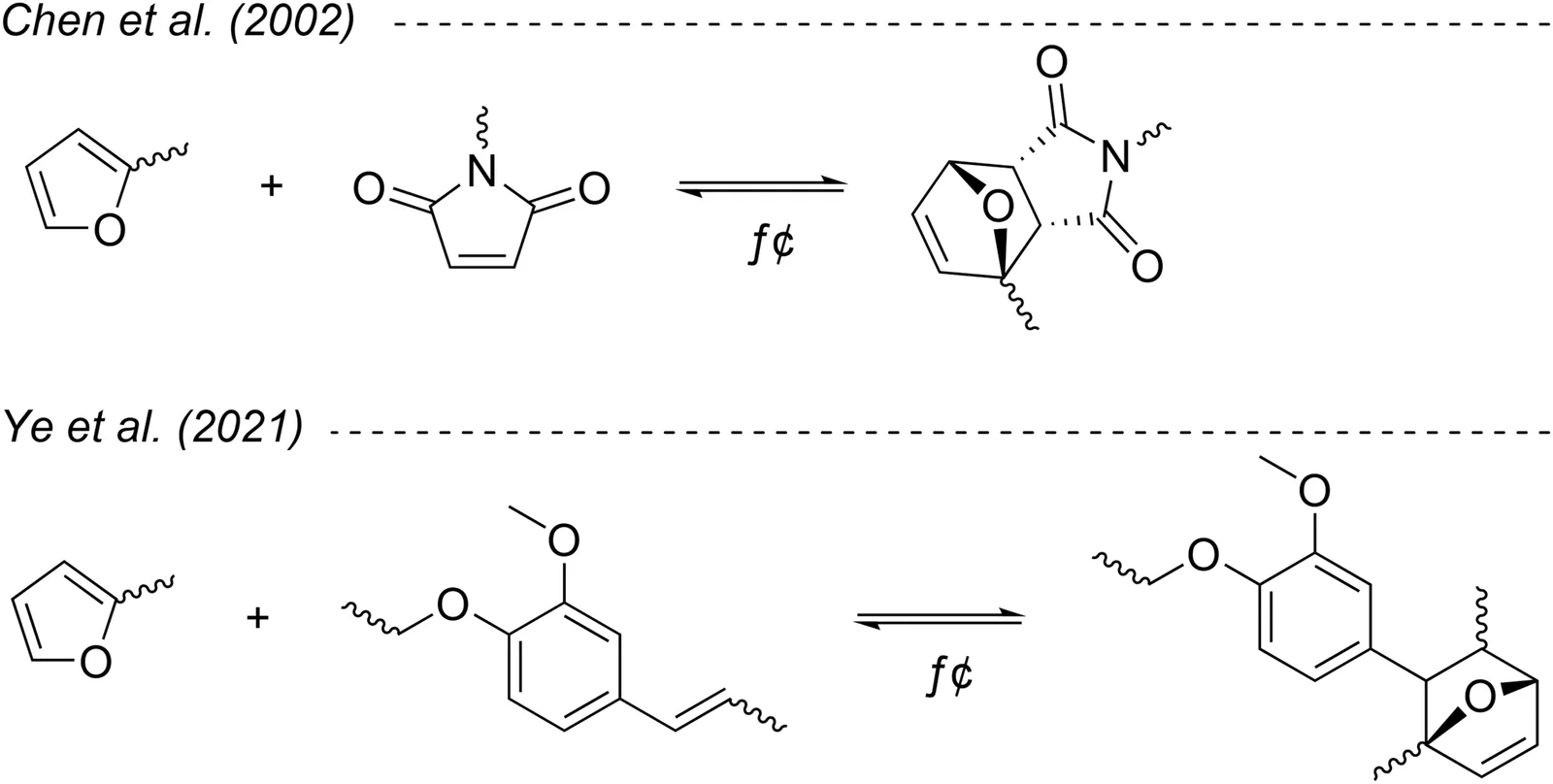
Diels–Alder cycloaddition of furanic moieties.

Furanic resins can also be prepared *via* the Diels–Alder cycloaddition with furanic moieties. The [4 + 2] cycloaddition between furan and maleimide has found significant use in self-healing coatings.^58^ First used to produce thermosets that could be recycled in 2002, the Diels–Alder reaction is favoured below 70 °C, whilst above 100 °C, the retro-Diels–Alder is the favoured reaction.^59^ It was demonstrated by Fang *et al.* that epoxy- and PU-based coatings based on a Diels–Alder reaction were able to show rapid self-healing and satisfactory mechanical and anti-corrosive properties.^60^ Transparent self-healing coatings were prepared from copolymers of furfuryl methacrylate and aliphatic bismaleimides using a similar method that were able to match the optical performance of polymethylmethacrylate (Fig. 10).^61^ This network was used to produce a self-healing luminescent solar concentrator, demonstrating the potential of this material in solar panels.^62^ The introduction of furan and maleimide groups to a poly(3,4-ethylenedioxythiophene) PU composite was shown to induce self-healing properties in a conductive material, although a careful balance is required, as a higher furan-maleimide content was found to decrease conductivity.^63^

Recently, this Diels–Alder framework has been used to produce self-healing resins for wood and glass coatings.^64^ Two low molecular weight DGEBA resins functionalised with furfuryl alcohol were prepared and cross-linked with bismaleimide. It was found that the resin that exhibited the better self-healing properties had poorer heat resistance and gloss retention. This framework has also been used with other dienophiles, such as ferulic acid, which can be derived from lignin. Epoxy monomers were prepared from furfuryl alcohol by Ye *et al.* (Fig. 10) and underwent subsequent cycloaddition with a ferulic acid-based epoxy network, allowing for co-crosslinking.^65^ The introduction of the furan-derived moiety was found to improve the mechanical properties of the resin, with the resin achieving a higher *T*_g_ (250 °C *vs.* 194 °C) and tensile strength (92.6 MPa *vs.* 82.5 MPa) compared to commercial DGEBA.

## 3. Vanillin

**Fig. 11:**
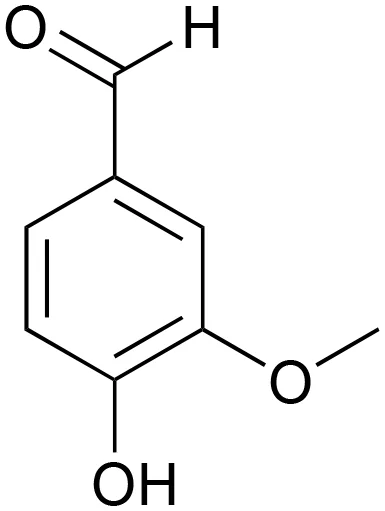
Vanillin (4-hydroxy-3-methoxybenzaldehyde).

Vanillin (4-hydroxy-3-methoxybenzaldehyde) (Fig. 11) is an aromatic plant-based metabolite bearing an aldehyde, methoxy, and aromatic alcohol functional group, which can be naturally derived from vanilla pods of the *Vanilla* species of orchids.^66,67^ However, due to the laborious process of harvesting vanillin from orchids, around 88% of global vanillin production is from petrochemical sources.^68^ The combination of the long petrochemical synthesis of vanillin in a catechol-guaiacol process and the high value of vanillin as a flavour and fragrance compound has led to its position as a “poster-child” of bio-based chemistry. There have been many approaches to deriving vanillin from cheaper biomass, with many biotechnological approaches to production being discovered and explored. Nevertheless, the cost of both approaches and the lack of technologies remain an issue.^69^

An emerging solution to this problem is the derivation of vanillin from lignin, the second most abundant bio-based polymer on earth, which is currently responsible for 11% of global vanillin production.^68^ The most common approach is the extraction of vanillin from lignin from paper production waste streams,^69^ although challenges remain due to the non-uniform structure of lignin and limited extraction methods.^70^ Nevertheless, vanillin has attracted attention as a platform molecule for resins in the coating industry due to its aromatic structure, diverse functional group handles, and its accessibility from biomass; and its application in bio-based aromatic polymers has been reviewed by Zhu *et al.*^71^

### 3.1 Polyurethane coatings

Vanillin has been widely incorporated into flame-retardant PU coatings to achieve high thermal and chemical stability due to its aromatic structure. The synthesis of monomers often relies on the condensation of the aldehyde handle with amines to form Schiff bases. The incorporation of a dynamic imine bond into the coating results in the formation of vitrimeric materials with self-healing properties. Furthermore, it extends coating recycling opportunities due to the presence of reversible cross-linkages, consistent with the principles of a circular chemical economy.

**Fig. 12:**
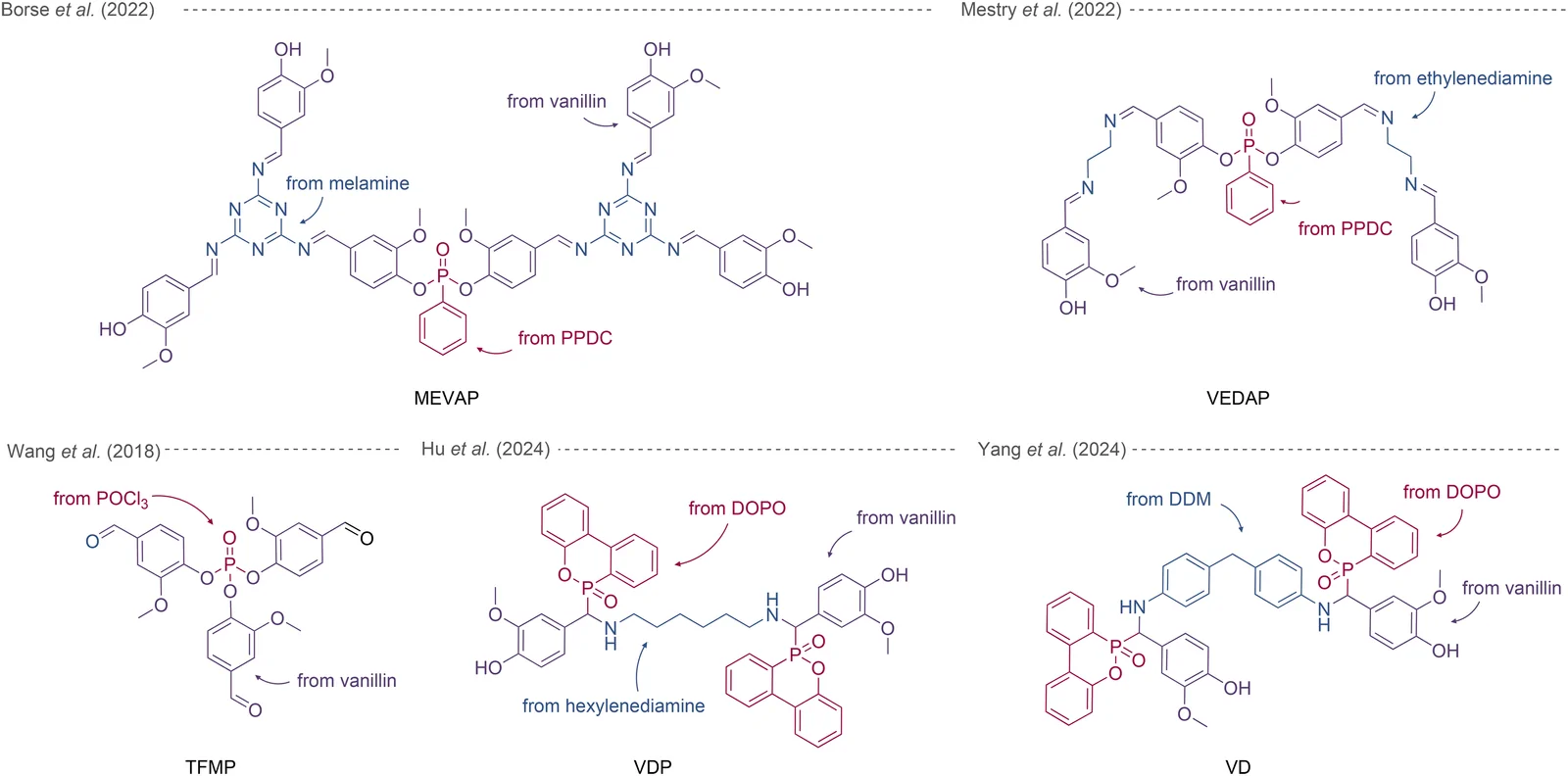
Vanillin Schiff base diol/tetraol monomers incorporating phosphorus in flame-retardant PU coatings. PPDC = phenyl-phosphonic-dichloride, VD = vanillin-based diol, VDP = vanillin-based diol containing organic phosphorus moieties, DOPO = 9,10-dihydro-9-oxa-10-phosphaphenanthrene-10-oxide, DDM = 4,4′-diaminodiphenylmethane, TFMP = tris(4-formyl-2-methoxyphenyl) phosphate.

Recent studies on vanillin-based PU coatings have focused on the introduction of a phosphorus species into the aforementioned Schiff bases to enhance flame-retardant properties. The pioneering work in the area was demonstrated by Wang *et al.* in the synthesis of covalent adaptable networks (CANs) by curing tris(4-formyl-2-methoxyphenyl) phosphate (TFMP) aldehyde (from vanillin and POCl_3_) with different diamines.^72^ The resultant thermoset exhibited high-performance characteristics and attractive recycling properties. More recently a similar concept was shown by Borse *et al.* and Mestry *et al.* in the development of diol/tetraol cross-linkers – melamine–vanillin–phosphorus (MEVAP) and ethylenediamine–vanillin–phosphorus (VEDAP), respectively (Fig. 12).^73,74^ These alcohols were combined with commercial PU-prepolymer and commercial polyols at different concentrations and coated onto mild steel panels for characterisation. An increase of MEVAP and VEDAP content improved thermal stability, flame retardancy, and degradation temperatures of the studied coatings. A similar concept was applied by Hu *et al.* and Yang *et al.* in the development of diol monomers, where the imine bonds, rather than vanillin's hydroxyl groups, were functionalised with a phosphorus species (Fig. 12).^75,76^

In addition to thermal stability and flame retardancy improvement, an increased content of these monomers in PUs enhanced mechanical properties of the polymers and influenced their morphology and glass transition temperatures (*T*_g_).

**Fig. 13:**
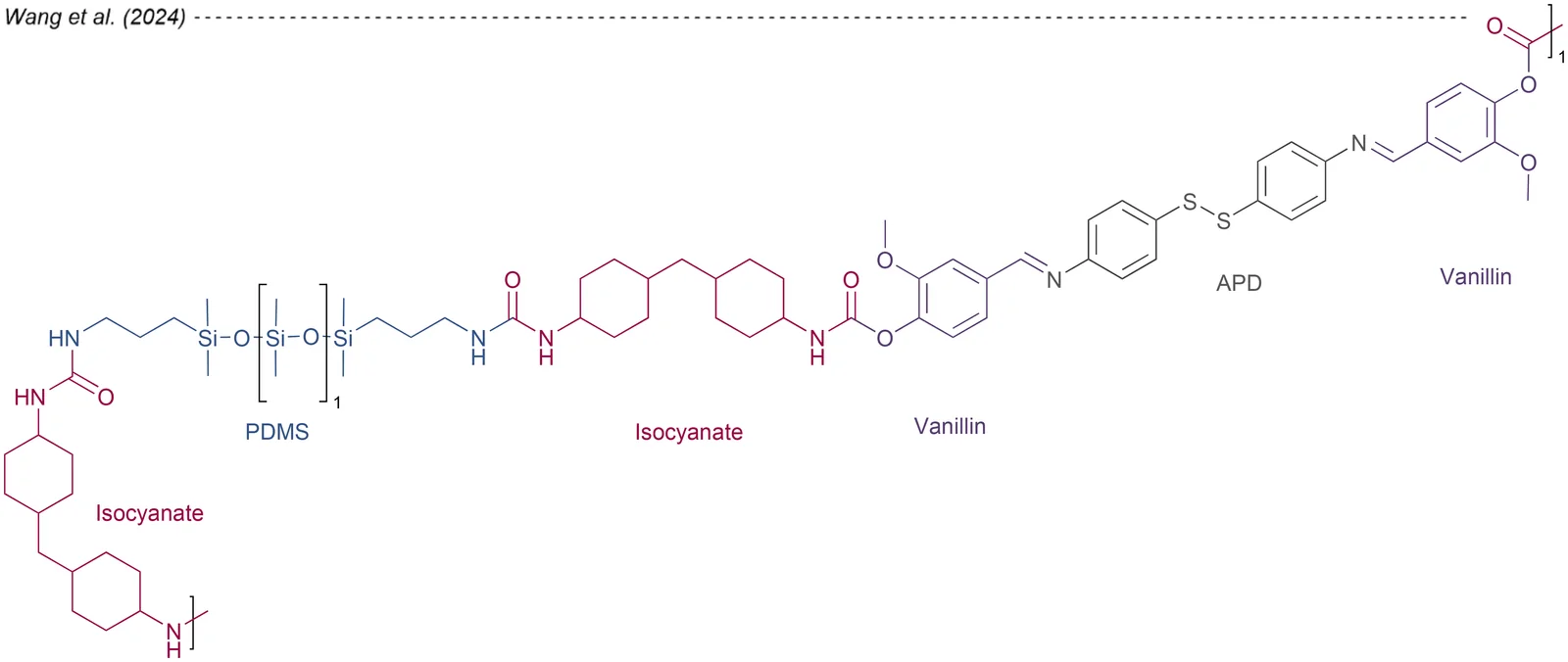
Vanillin Schiff base ice-phobic PU coating with silicone oil. PDMS = polydimethylsiloxane, APD = 2-aminophenyl disulfide.

The applications of vanillin Schiff base PUs extends to ice-phobic coatings. Wang *et al.* reported vanillin-based polyols terminated with isocyanates based on silicone oil to lower ice adhesion, increase hydrophobicity and improved anti-fouling (Fig. 13).^77^ The resulting coating exhibited UV-shielding and self-healing properties in extreme environments.

**Fig. 14:**
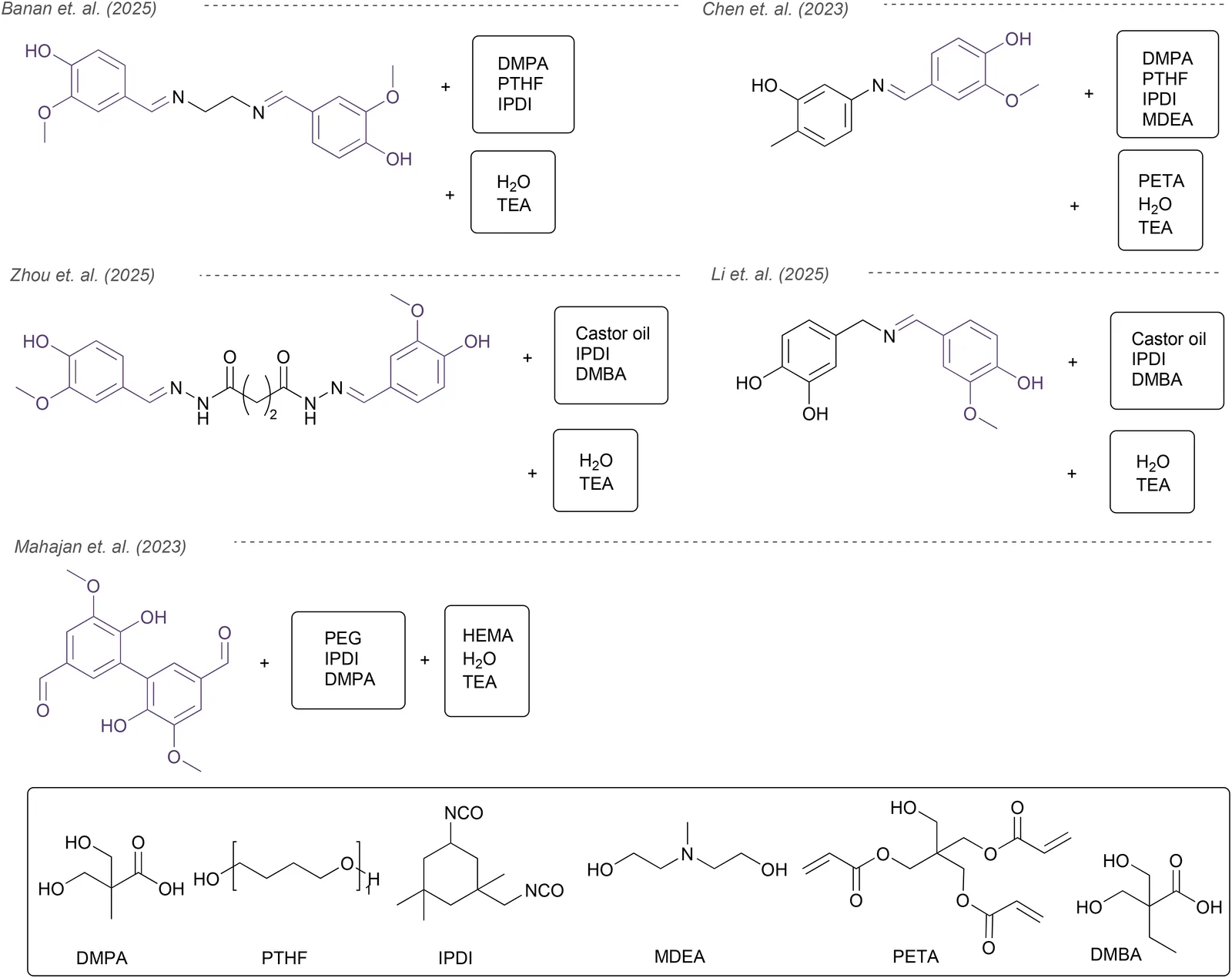
Vanillin Schiff base diol monomers for WPUs. HEMA = hydroxyethyl methacrylate, TEA = triethylamine, PEG = poly(ethylene) glycol, DMPA = dimethylolpropionic acid, PTHF = polytetrahydrofuran, IPDI = isophorone diisocyanate, MDEA = methyldiethanolamine, PETA = pentaerythritol tetraacrylate, DMBA = dimethylol butanoic acid.

WPUs attract attention as an environmentally friendly alternative to resins dispersed in organic solvents. However, poor long-term durability and mechanical properties of WPUs remains an issue. The incorporation of vanillin Schiff bases can address this problem by both improving mechanical properties and introducing self-healing capabilities of these polymers. This concept was demonstrated by Banan *et al.* and Chen *et al.* yielding a self-healing coating with improved tensile strength and broadened recycling opportunities (Fig. 14).^78,79^ Additionally, an interesting research direction emerges by utilising the aforementioned idea in the context of WPUs incorporating castor oil (CWPU). The incorporation of castor oil into the coating increases its bio-based content, serving as an alternative for petroleum-based polyols, but reduces its tensile strength due to the introduction of soft aliphatic backbones. Zhou *et al.* addressed this challenge by incorporating novel vanillin-succinohydrazide-based Schiff base diols into CWPU coatings (Fig. 14).^80^ The introduction of an imine bond induced self-healing properties and increased the tensile strength, as well as providing a handle for recycling. Further improvement to self-healing CWPUs has been made by Li *et al.* through the incorporation of vanillin-derived Schiff base monomers and graphene oxide (GO) nanosheets which enhance the anti-corrosive properties (Fig. 14).^81^ Lastly, Mahajan *et al.* used divanillin as a diol monomer in the synthesis of UV-curable coatings functionalised with methacrylates for wood coating applications (Fig. 14).^82^

**Fig. 15:**
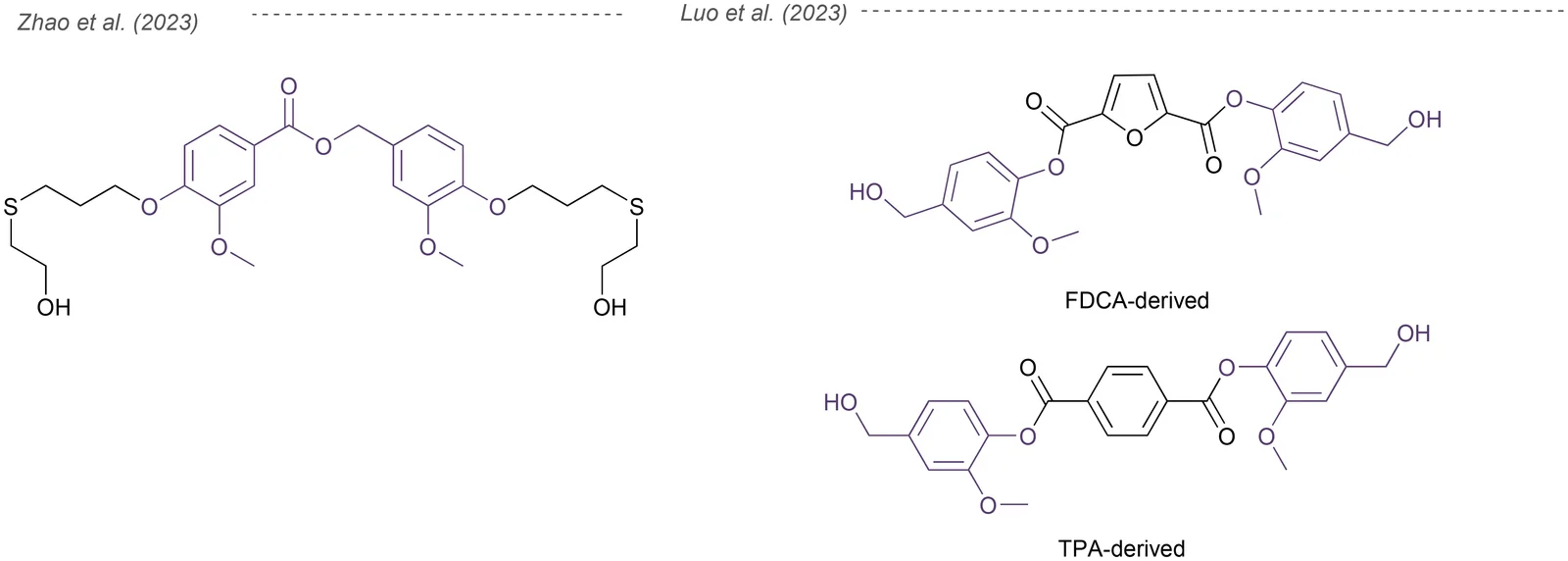
Esters in vanillin-based diol monomers for biodegradable PU coatings. TPA = terephthalic acid.

Degradability of commercial PU thermosets remains a challenge, which can be tackled by the incorporation of dynamic imine bonds in the coating structure as demonstrated above (Fig. 14). Zhao *et al.* showed a different approach by introducing an ester bond between two vanillin molecules in the diol monomer, serving as a biodegradation handle (Fig. 15).^83^ The resulting coating had over 70% bio-based carbon content and exhibited comparable mechanical properties to commercial thermosetting PUs.^83^ The researchers have also demonstrated a full degradation of the invented resin within few hours under basic conditions. A similar approach was demonstrated by Luo *et al.* where ester bonds were incorporated into the diol monomers to yield degradable PU coatings with encouraging mechanical properties, thermal stability and solvent resistance (Fig. 15).^84^ The above studies demonstrate a recent interest in new strategies for more efficient polymer degradation and the sourcing of polymer components from bio-based materials. This answers the need of transforming the chemical industry into a circular economy.

**Fig. 16:**
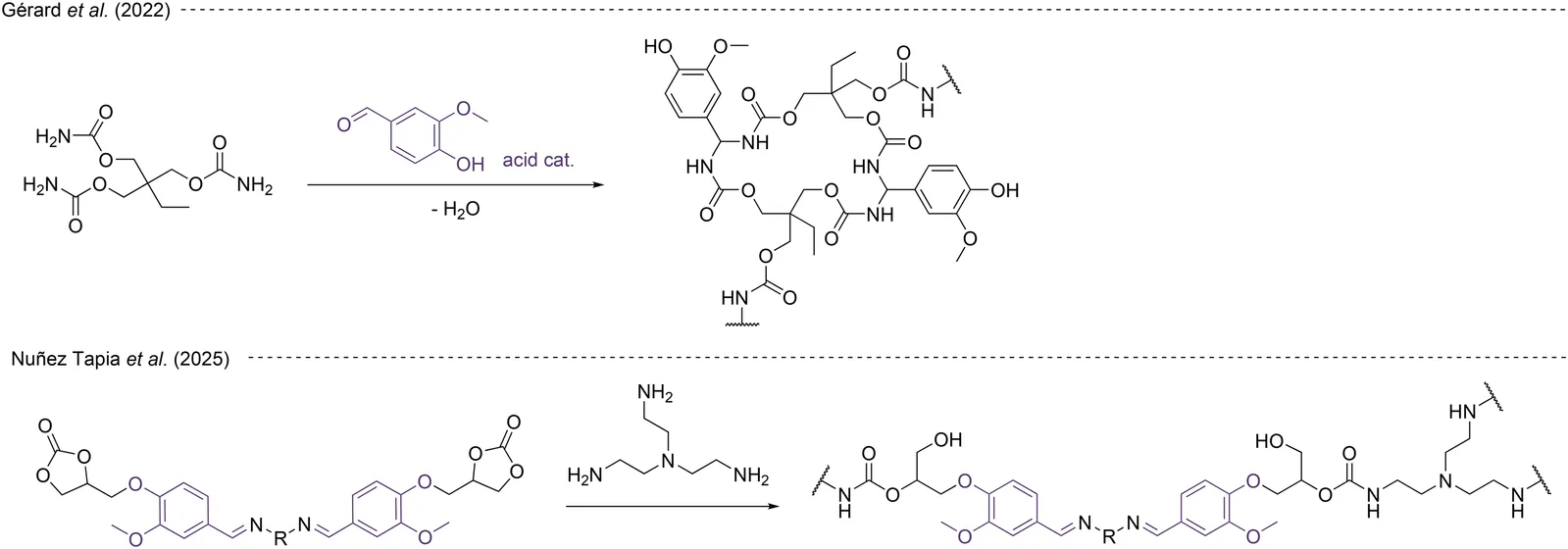
Synthetic strategies for NIPU synthesis demonstrated on vanillin.

Another key challenge associated with PUs is that the synthesis of many polyisocyanates has traditionally involved toxic reagents such as phosgene, which is inherently incompatible with the 3^rd^ of the 12 Principles of Green Chemistry (less hazardous synthesis). Different PU preparation strategies, such as non-isocyanate PU (NIPU), are currently being investigated.^85^ One of the synthetic ideas suggested by Gérard *et al.* relies on the condensation of di- and tri-carbamates with bioderived aldehydes, including vanillin, avoiding the need for polyisocyanates as a precursor (Fig. 16).^86^ Nevertheless, the synthesis optimisation still presents major challenges. Another approach was undertaken by Nuñez Tapia *et al.*, developing non-isocyanate polyhydroxyurethane (NIPHU) coatings *via* the polycondensation of vanillin cyclic carbonate Schiff base monomers with triamines (Fig. 16).^87^ The synthesised PU exhibited self-healing properties and recyclability.

### 3.2 Epoxy coatings

**Fig. 17:**
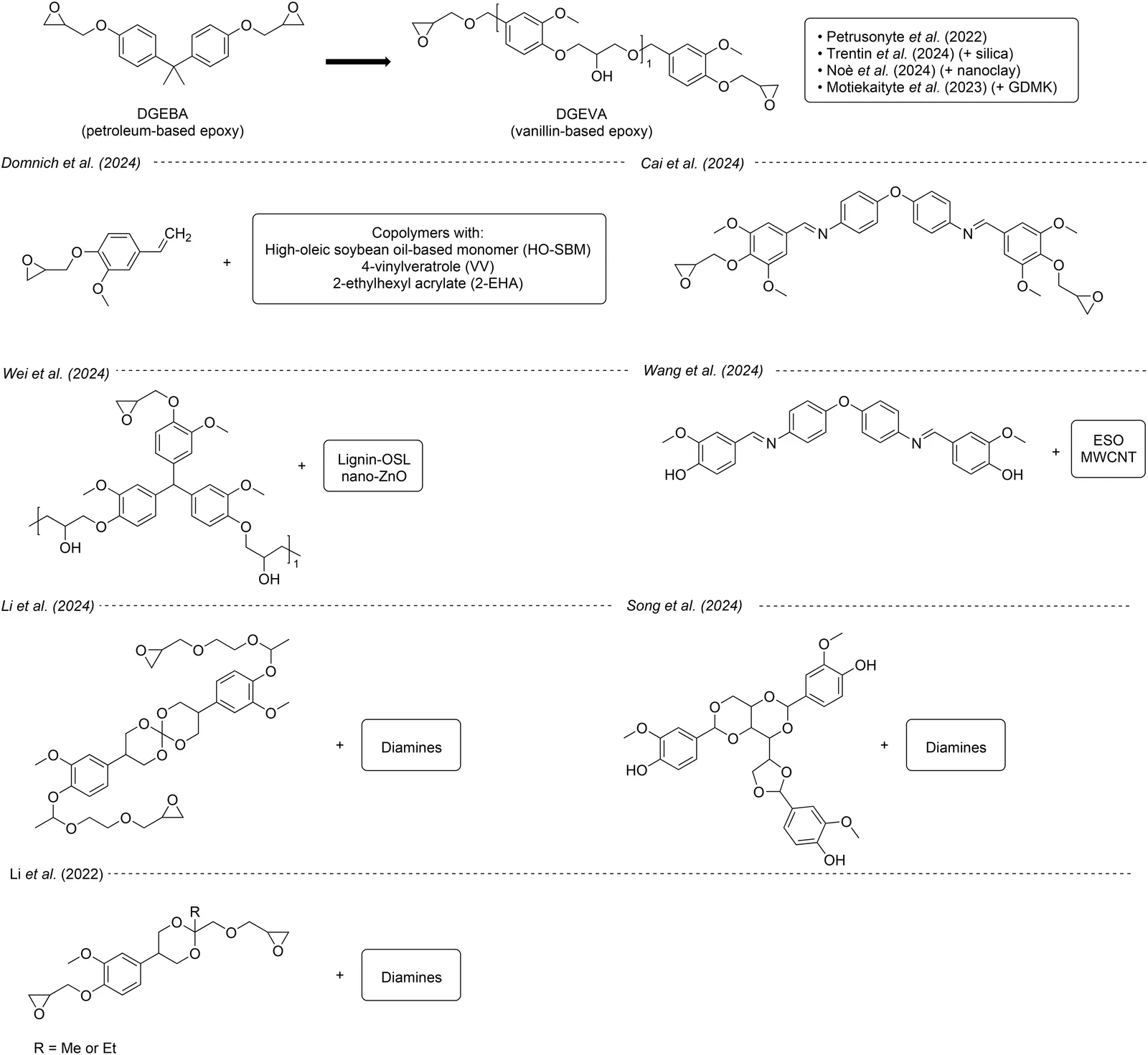
Vanillin-based monomers incorporated in epoxy coatings. OSL = octadecyltrichlorosilane.

Research in the area of vanillin-based epoxy coatings addresses bio-based content and recycling strategies of new resins. The majority of studies focus on anti-corrosive, anti-microbial properties improvement and long-term durability. This is commonly achieved by incorporating various nanomaterials into the composites containing vanillin-based epoxides which further enhances adhesive and mechanical properties by tightening the protective layer. Vanillin alcohol diglycidyl ether (DGEVA) has been developed as a bio-derived alternative to DGEBA for epoxy resins. DGEVA has been utilised in UV-curable epoxy resins, which require less energy to cure compared to the thermally cured counterparts (Fig. 17).^88^

Trentin *et al.* have further functionalised DGEVA resin with silica nanoparticles achieving high adhesion, thermal stability and anti-corrosive properties (Fig. 17).^89^

Noè *et al.* developed a DGEVA-based UV-curable epoxy resin for anti-corrosive coatings, utilising nanoclay as a bio-derived alternative to commonly used GO nanoparticles (Fig. 17).^90^ The coating exhibited satisfying properties while tackling the common problem of GO aggregation in the formulation.

A triphenyl vanillin-guaiacol epoxy resin was prepared by Wei *et al.* incorporating lignin modified with octadecyltrichlorosilane (OSL) to achieve a low surface energy coating with superhydrophobic (SHP) properties (Fig. 17).^91^ The incorporation of zinc oxide nanoparticles into the composition lowered the roughness of the coating and increased its anti-corrosive properties. The approach presented by the group not only introduces a new bio-based resin design with excellent properties upon nanoparticle addition but also demonstrates the possibility of direct lignin valorisation.

Motiekaityte *et al.* copolymerised DGEVA with glycerol dimethacrylate (GDMA) to develop a coating cured *via* a dual free radical and cationic photopolymerisation process (Fig. 17).^92^ Different ratios of monomers yielded coatings with different properties – increasing the acrylate content resulted in higher mechanical performance, while increasing the epoxy vanillin content gave greater antimicrobial properties. Dual-curing processes were also studied by Domnich *et al.* on resins with acrylic monomers of highly oleic soybean oil and vanillin-derived epoxy monomers.^93^ Epoxy resins were cured by the addition of different diamines, autooxidation of allylic groups, or both in a dual-curing process. Epoxy resins cured by amines, as well as by dual-curing, exhibited denser networks compared to autooxidation-cured resins.

Epoxy coatings incorporating vanillin Schiff base have also been studied by Cai *et al.* who reported a flame-retardant coating^94^ and Wang *et al.* who developed SHP coatings from epoxidised soybean oil (ESO) and a vanillin-derived imine-based curing agent to enhance self-healing properties and recyclability (Fig. 17).^95^ Multi-walled carbon nanotubes (MWCNTs) were further introduced to enhance the hydrophobic properties and de-icing performance of the coating in the latter study.

Other nanomaterials used to tune the properties of epoxy resins include MXene nanosheets (two-dimensional inorganic layers) coated with *o*-vanillin-based zeolitic imidazolate frameworks combined with epoxy resins to improve flame-retardant properties,^96^ and GO grafted with vanillin in epoxy vinyl ester resins to improve their anti-corrosive properties^97^

An interesting research direction emerges in the efforts to advance degradability of thermosetting epoxy resins especially in the context of carbon fibre reinforced polymers (CFRPs). Valuable studies have been delivered by Li *et al.* in the synthesis of spiro diacetal epoxy monomers.^98^ Vanillin was condensed with pentaerythritol and the product was substituted with 2-(vinyloxy)ethanol and epichlorohydrin to deliver epoxy monomers cured with diamines. The resulting material showcased faster degradation under mild acidic conditions compared to previously reported polymers. Furthermore, carbon fibres were fully recycled from the degraded epoxy monomer demonstrating reprocessability of these composites. The processibility of acetal-based epoxy monomers after degradation has also been investigated to deliver monomers of lower viscosity and comparable mechanical properties, recyclability and carbon fibre recovery as demonstrated by Li *et al.*^99^ A similar acetal-based monomeric scaffold was also reported by Song *et al.* to advance mechanical and thermal properties of the aforementioned class of polymers.^100^ The proposed novel acetal-based triol curing agent for DEGEVA epoxy was synthesised from the three vanillin units and sorbitol. The resulting material exhibited good properties and high recyclability under mild conditions.

### 3.3 Epoxy curing agents

**Fig. 18:**
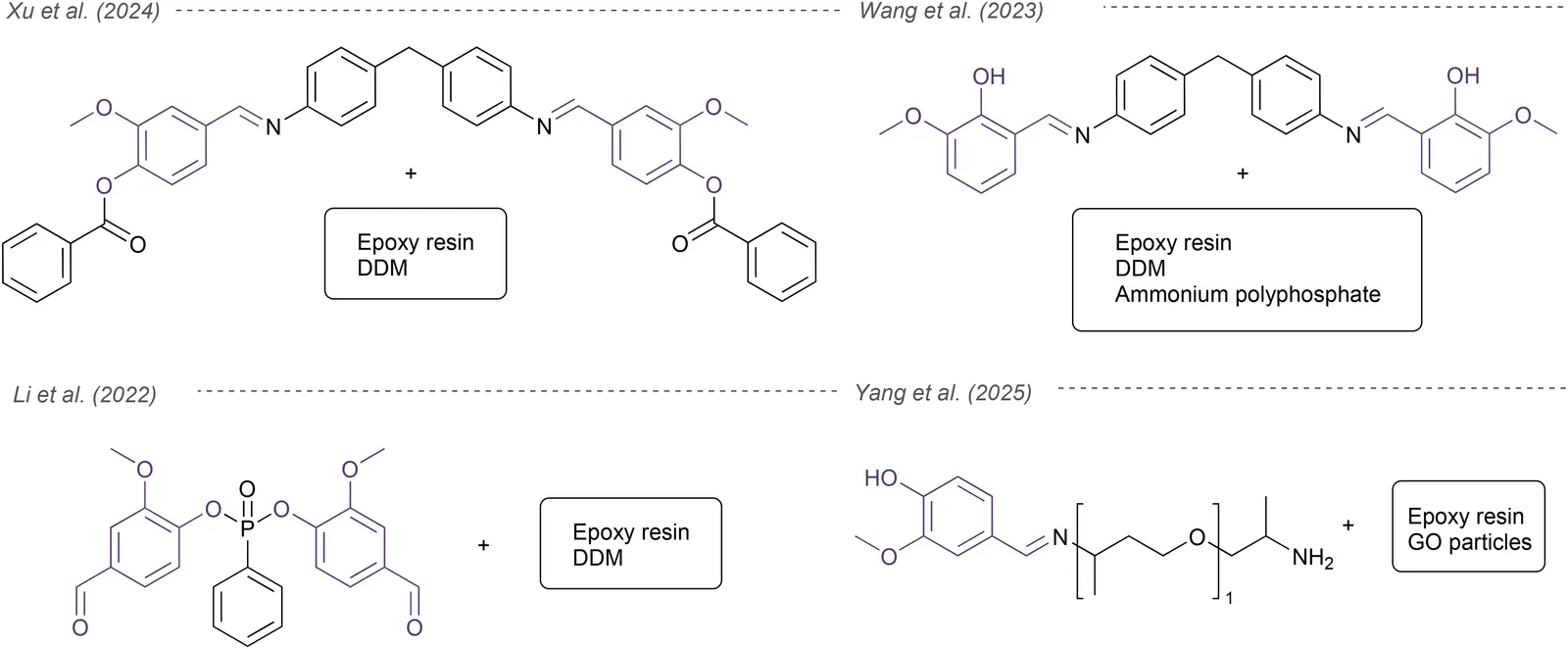
Vanillin-based curing agents for epoxy resins. DDM = 4,4′-diaminodiphenylmethane.

Vanillin Schiff bases have been studied as an alternative diamine curing agent to introduce self-healing properties to epoxy coatings (Fig. 18).^101^ Many studies combined this idea with the introduction of a phosphorus species to enhance flame-retardant properties of the developed coatings (Fig. 18).^102,103^ Nanotechnology has also been introduced. For example, Liang *et al.* designed a Schiff base curing agent from vanillin and nanoparticles consisting of –Si–O–Si– chains functionalised at the peripheries with organic functional groups.^104^ The resulting intermediate was further functionalised with an organophosphorus species to yield a self-healing, flame-retardant, and UV-protective coating for wood. In a similar study, Yang *et al.* treated commercial epoxy resins with curing agents made from vanillin and polyester amine D230 to form self-healing coatings for marine environment applications (Fig. 18).^105^ The developed coating incorporated GO to further enhance anti-corrosion and aging resistance.

### 3.4 Polyester coatings

Polyesters derived from vanillin have been previously considered as a bio-based replacement for poly(ethylene terephthalate) (PET).^106–108^ Many studies focused on the improvement of existing vanillin-based polyesters to achieve better properties and simplified greener syntheses.^109–112^

**Fig. 19:**
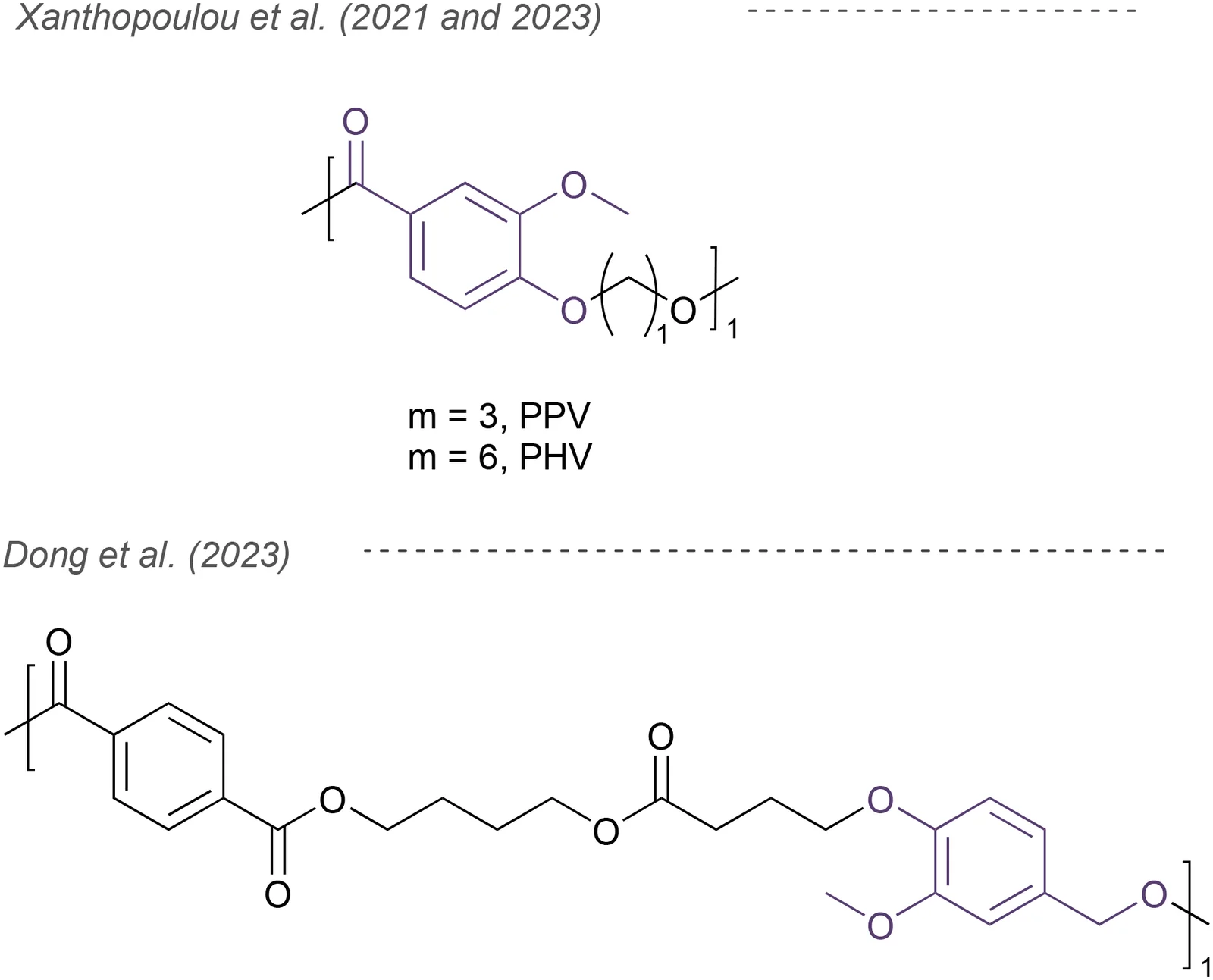
Recent advances in vanillin-derived polyesters.

More recently, the first syntheses and characterisation of poly(propylene vanillate) (PPV) and poly(hexylene vanillate) (PHV) were reported as PET alternatives (Fig. 19).^113,114^ Notably, PHV exhibited thermoplastic elastomeric behaviour and was found to be fully processable at temperatures above 50 °C, allowing for lower energy processing compared to other alipharomatic‡‡Polyester derived from aliphatic diols, such as furfural or hydroxymethylfurfural. polyesters.^114^

A recent study by Dong *et al.* demonstrated how vanillin and its oxygen-containing functional groups can overcome the challenge of the limited strength and recyclability of bio-derived hot-melt adhesives.^115^ The oxygen-containing functional groups of vanillin can interact with positively charged surfaces like metals, increasing the adhesion strength. Partially bio-based copolyesters were synthesised with varied vanillin-monomer content (Fig. 19), and the optimised thermoplastic copolyester exhibited a high level of adhesion and recyclability, as well as resistance to heat, acids and alkalis.

### 3.5 Polyacrylate coatings

**Fig. 20:**
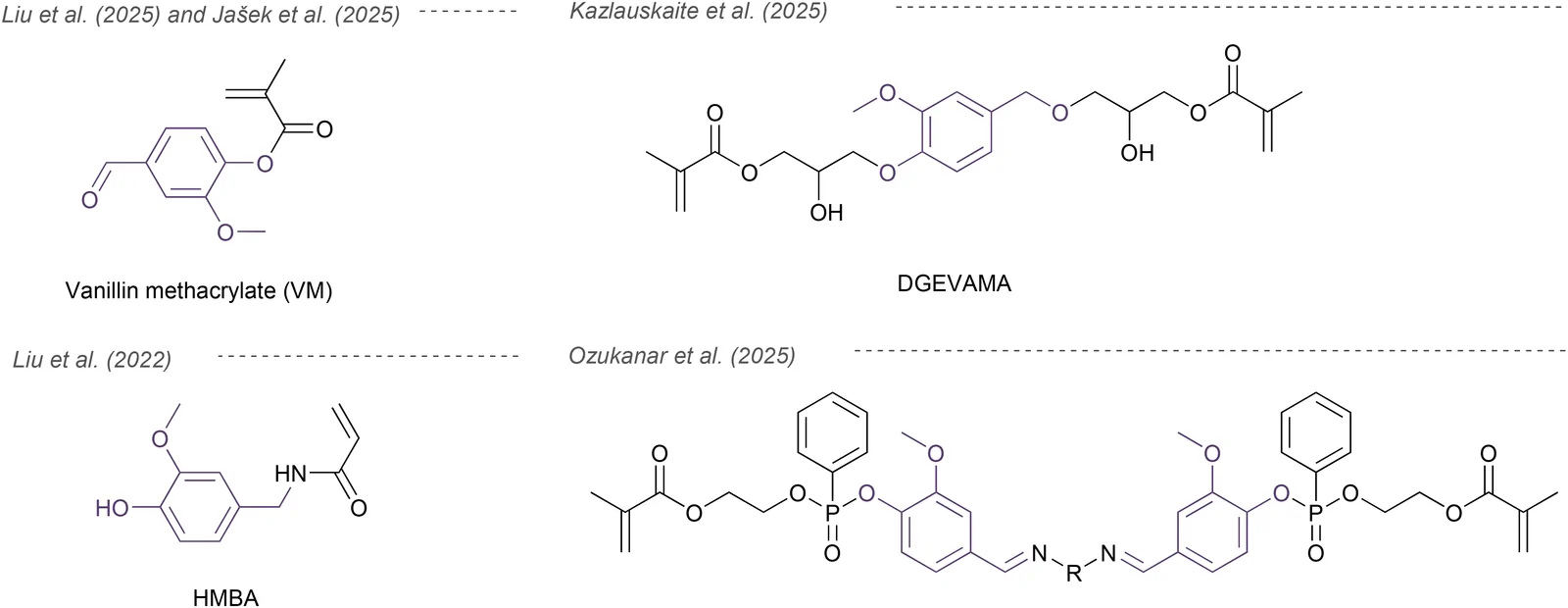
Vanillin incorporated into polyacrylates. DGEVADMA = vanillin alcohol diglycidyl ether dimethacrylate, HMBA = *N*-(4-hydroxy-3-methoxybenzyl)-acrylamide.

Polyacrylates represent a more niche field of vanillin-containing coatings. Nevertheless, vanillin has been incorporated into polyacrylates for several applications. Firstly, as an environmentally friendly alternative for heavy metal-based anti-fouling agents in marine self-polishing coatings (Fig. 20).^116,117^ Similarly, due to vanillin's antimicrobial properties, vanillin and glycerol were employed by Kazlauskaite *et al.* in the development of thiol-based polyacrylate self-healing coatings (Fig. 20).^118^ Vanillin-containing Schiff base dimethacrylate monomers with phosphorus species were also developed by Ozukanar *et al.* for anti-corrosive coil coatings (Fig. 20).^119^ Lastly, vanillin methacrylate (VM) and methacrylated vegetable oil have been used in bio-based UV-shielding coatings for wood with good adhesion properties (Fig. 20).^120^

### 3.6 Benzoxazine coatings

**Fig. 21:**
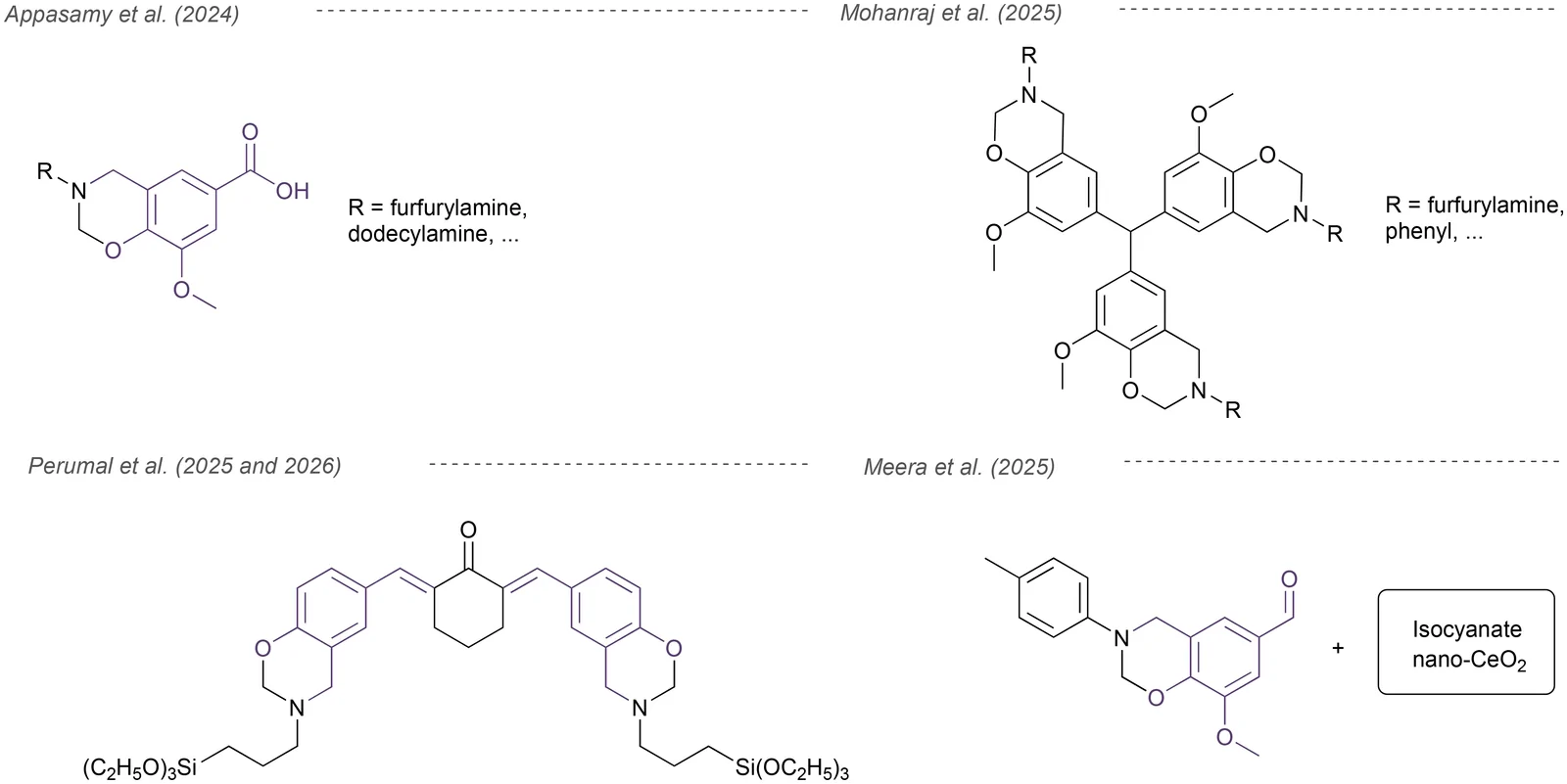
Vanillin-based benzoxazine monomers.

The synthesis of benzoxazine monomers from vanillic acid often incorporates other xylochemicals, like furfurylamine, to develop bio-based coatings for hydrophobic, anti-corrosive and thermally resistant applications (Fig. 21).^121,122^ Recent studies by Perumal *et al.* have contributed superhydrophobic polybenzoxazine coatings functionalised with silica for anti-corrosive and oil–water separation properties (Fig. 21).^123,124^ Polybenzoxazine-PU copolymers were also prepared by Meera *et al.* with cerium oxide incorporated into the coating to yield coatings with enhanced anti-corrosive properties (Fig. 21).^125^

### 3.7 Other coatings

**Fig. 22:**
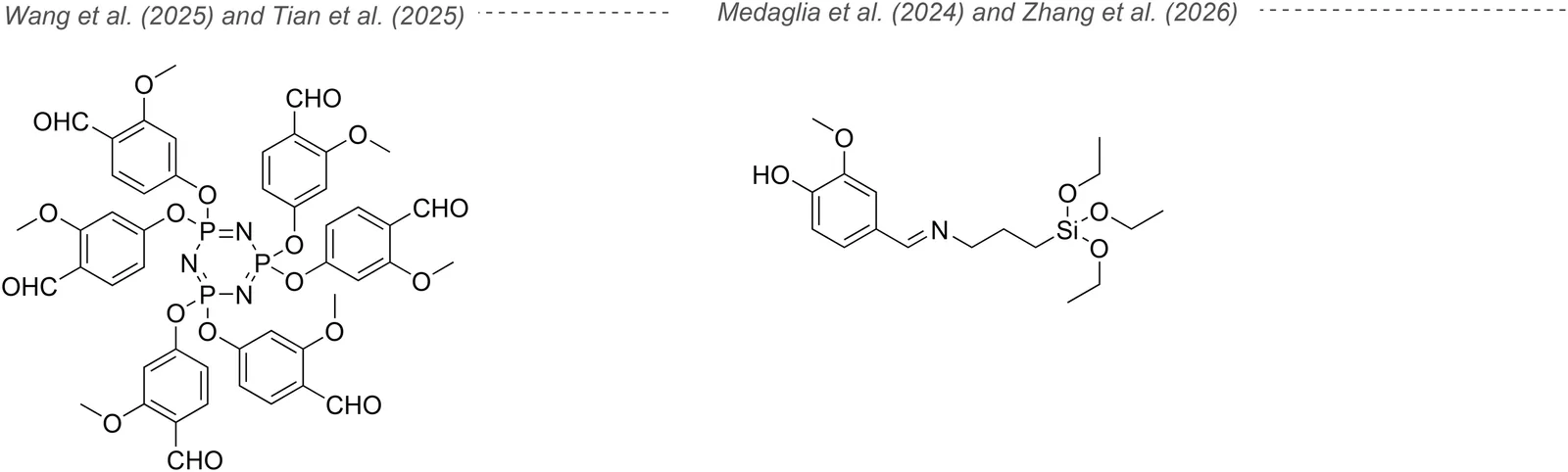
Vanillin cross-linked by phosphazene and functionalised with aminosilanes.

As previously mentioned, due to the presence of aldehyde functionality, vanillin-based polyimides have been extensively explored in recent years. Hyperbranched polyimides with vanillin-based phosphazene moieties were designed by Wang *et al.* yielding ice-phobic, UV-shielding, self-healing and flame-retardant coatings.^126^ The use of phosphazene as a cross-linker to enhance high-performance properties like flame-retardancy, high thermal and mechanical stability has been recently demonstrated by Tian *et al.* as well (Fig. 22).^127^ Vanillin hydroxyl groups were reacted with phosphazene to form aldehydes further condensed with aminoalcohols for their incorporation into PUs. The resulting coatings exhibited desired properties and remarkable reprocessability with high bio-based content. Functionalisation of aminosilanes with vanillin loaded on silica (nanoparticles, layers, dendritic) has also been explored recently for anti-corrosive applications.^128–130^

## 4. Cardanol

Cardanol is a phenolic lipid that can be extracted from Cashew Nut Shell Liquid (CNSL). CNSL is a by-product of the cashew industry and comes from the inedible shell of the nut. The cashew tree, *Anacardium occidentale* L., is native to Brazil but is now grown in multiple tropical countries including India, Mozambique, and the Philippines.^8^ The global market for cardanol was around $32 million in 2022, and is expected to rise to $58 million by 2028, with a global production of around 800 000 tonnes per year.^131^

Traditionally, CNSL was extracted from the shell either through a hot oil process or by roasting,^8^ but modern methods include solvent extraction or supercritical CO_2_ extraction.^132^ These different extraction processes result in different compositions of the CNSL, but all result in impurities which include sulfides, nitrogenous materials and minerals. Acid treatment of the CNSL, with aqueous hydrochloric acid or sodium hydrogen sulfate has been shown to reduce the number of impurities.^8^

**Fig. 23:**
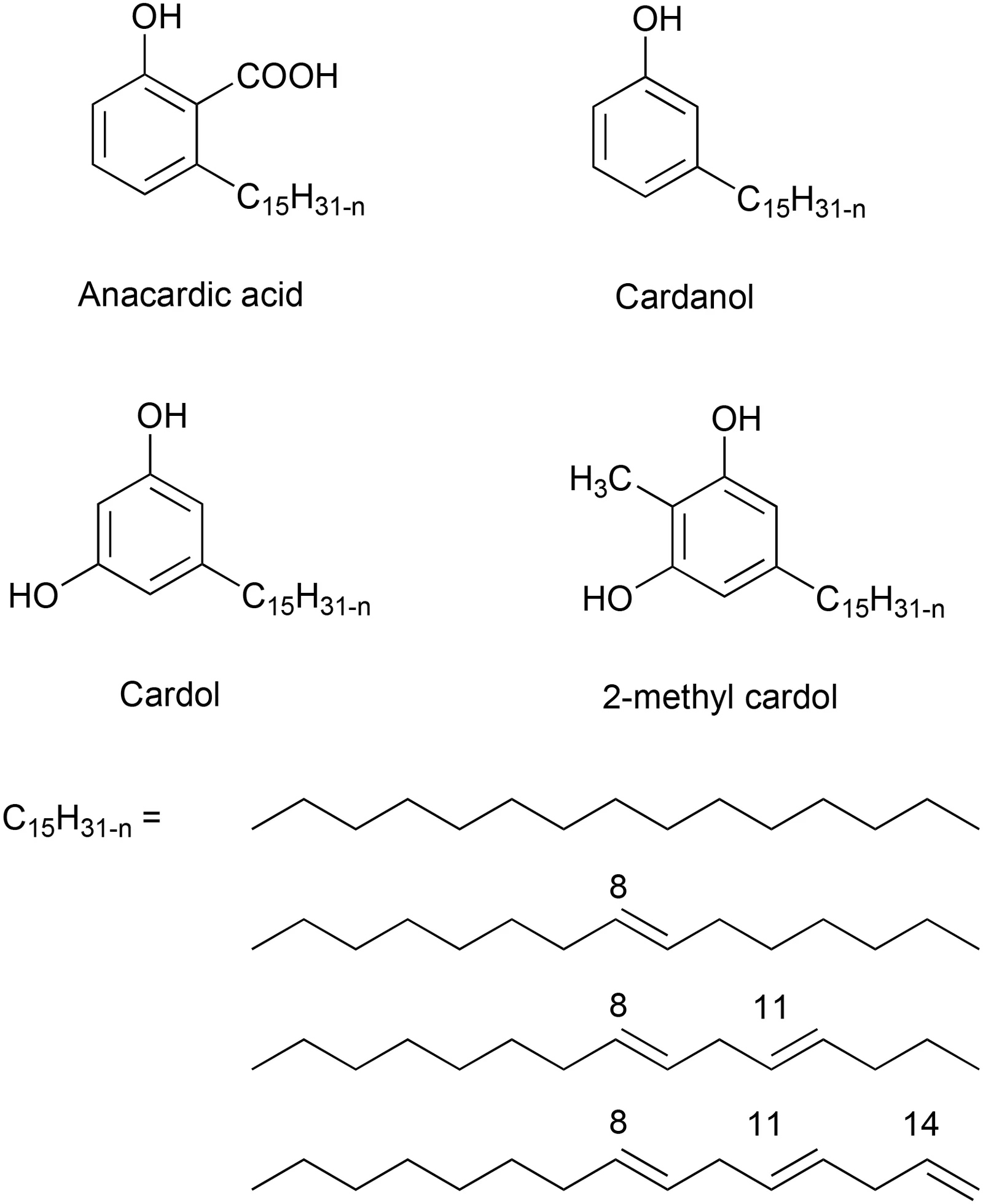
Structures of the compounds extracted from cashew nut shell liquid.

CNSL has four main components, anacardic acid, cardanol, cardol and 2-methyl cardol. In natural CNSL, the largest component is anacardic acid, however this decarboxylates to cardanol upon heating in treatment processes, meaning that cardanol is the main component in industrially processed CNSL.^133^ Cardanol itself is not one single compound, but a mixture of compounds with differing levels of unsaturation in the alkyl chain (Fig. 23). The unsaturation is found at the 8^th^, 11^th^ and 14^th^ carbon atoms, for increasing levels of unsaturation.

One major application of cardanol is as a phenol substitute, but the long alkyl chain brings additional hydrophobicity. Cardanol can be polymerised in a variety of different ways, as it has multiple reactive sites: the C=C bonds in the alkyl side chain, the phenol group, and the aromatic ring itself. This functionality grants access to a wide range of polymers and resins, including PUs, phenalkamines, phenolic resins, and epoxy resins, all of which are utilised in the paints and coatings industry, and were reviewed in depth by Voirin *et al.* in 2014.^134^

### 4.1 Epoxy coatings

Cardanol has several sites that can be epoxidised, including the phenolic OH, and the double bonds within the alkyl chain. These epoxides can then be ring-opened by amines to form epoxy resins. One example of this is the reaction of cardanol with epichlorohydrin to form an epoxidised cardanol^135^ which can then be used as an alternative to BPA.^136^ This epoxidised cardanol is already commercially available from Cardolite.^137^

**Fig. 24:**
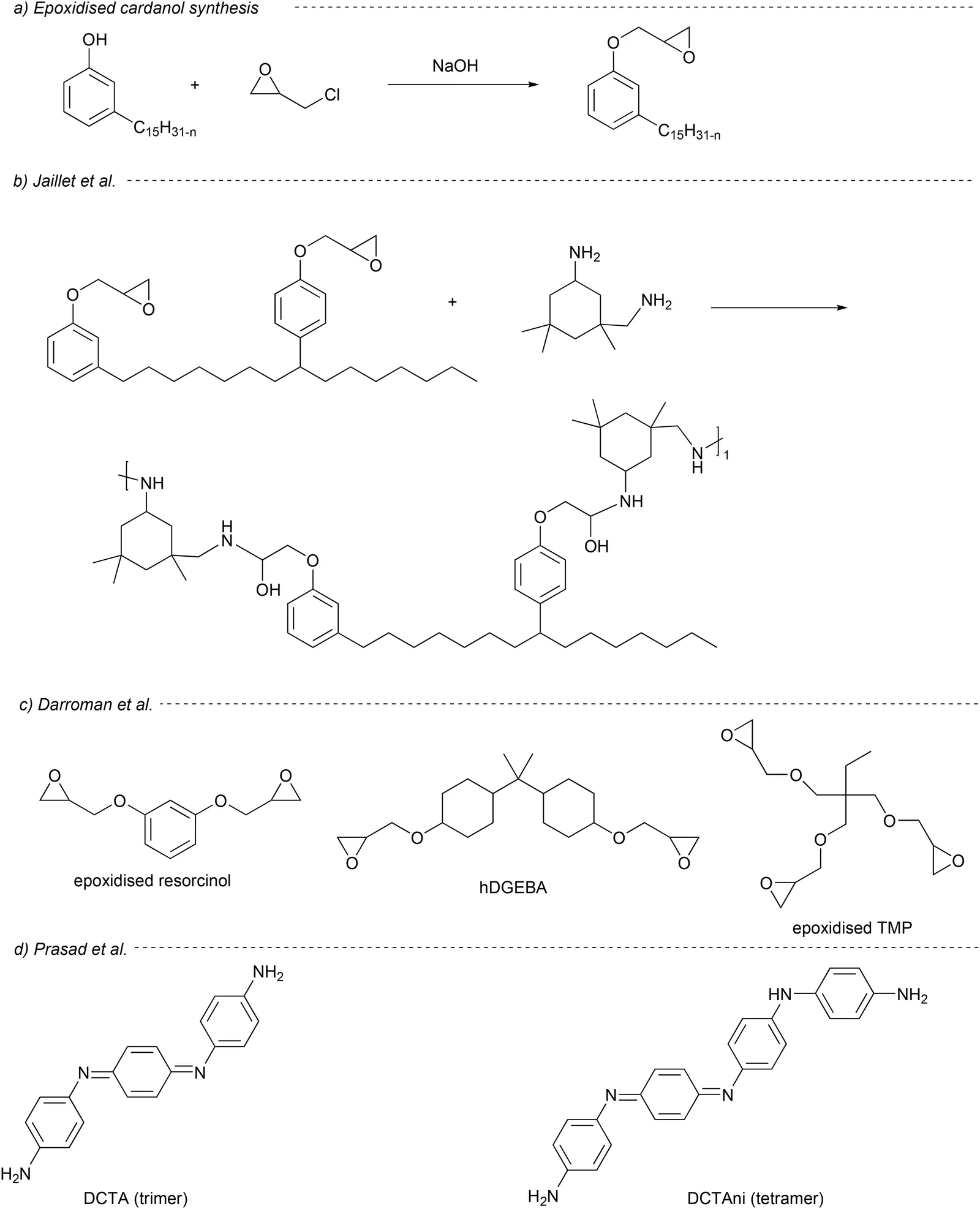
(a) Synthesis of epoxidised cardanol. (b) Epoxy resin synthesis. (c) Epoxy resin additives. (d) Oligoaniline curing agents.

Previous research by the Caillol group reacted the epoxidised cardanol with isophorone diamine and Jeffamine D400 diamine to form an epoxy resin. They compared this to a typical epoxy resin formed with DGEBA.^138^ While the cardanol-based resin had good properties, it did not match those of DGEBA. Further work utilised three reactive epoxy additives, resorcinol, hydrogenated DGEBA (hDGEBA) and trimethylolpropane (TMP), to improve the properties of the cardanol epoxy resin (Fig. 24).^139^ Resorcinol and hDGEBA both improved the *T*_g_ and hardness of the resin, whereas the addition of TMP improved the thermal stability of the resin. Utilising these additives produced a resin with more comparable properties to a typical epoxy resin formed from DGEBA. The effect of varying bio-based content on the mechanical characterization of cardanol epoxy resin blends has been explored by Iadarola *et al.*^140^

More recent research from Prasad *et al.* utilised aniline oligomers such as amine-capped trimer (DCTA) and tetramer (DCTAni) as the curing agents for epoxidised cardanol, along with 20 wt% DGEBA and butanediamine to give enhanced thermal, mechanical and anti-corrosion properties.^141^

**Fig. 25:**
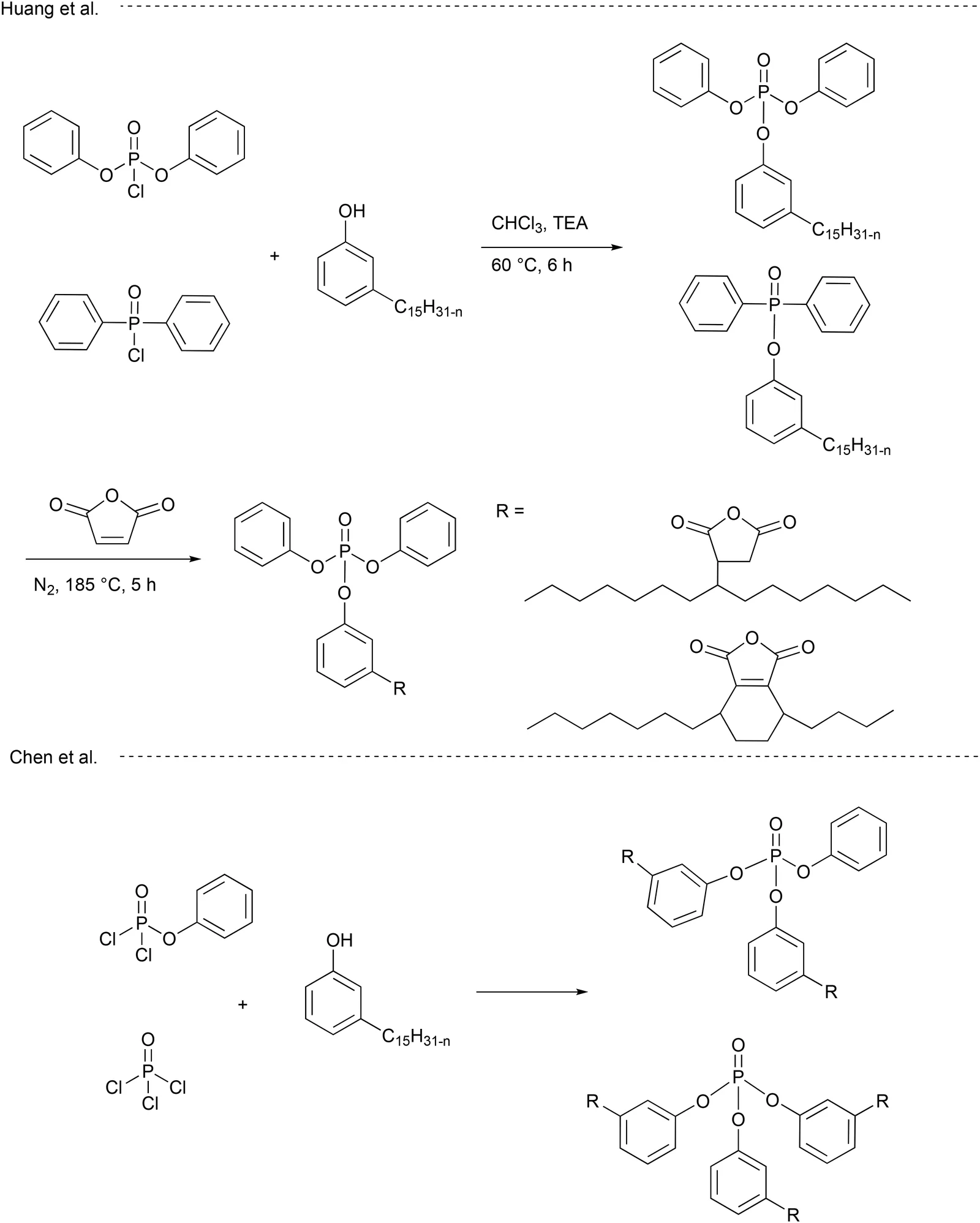
Flame retardant cardanol based curing agents.

Another recent advancement is the use of phosphorus containing curing agents to enhance the flame-retardant properties of cardanol-based epoxy coatings. Huang *et al.* used diphenyl chlorophosphate (DCP) and diphenylphosphinic chloride, reacting them with cardanol and maleic acid to form cardanol based curing agents (Fig. 25).^142^ These were then reacted with DGEBA to form epoxy resins which demonstrated enhanced flame-retardancy when compared to a conventional epoxy resin. The resins also had high thermal stability and good mechanical properties. Chen *et al.* also used DCP, as well as phosphorus oxychloride, and phenyl dichlorophosphate (PDP). Again, they reacted them with cardanol and maleic acid to form cardanol based curing agents.^143^ These were then reacted with DGEBA to form epoxy resins which also demonstrated enhanced flame-retardancy when compared to a conventional epoxy resin.

These methods still employ petrochemically derived BPA monomers, so whilst the bio-based content is increased by using some cardanol, it is not fully bio-based. Wang *et al.* improved upon this using a cardanol epoxy monomer along with a cardanol-derived phosphate linker, to produce another epoxy resin, with good flame-retardancy, thermal stability and mechanical strength, that also has a higher bio-based content.^144^ This epoxy resin has a much higher bio-based content compared to just using a cardanol-based phosphate crosslinker, however the sustainability credentials of this polymer are adversely impacted by the synthesis of the cardanol epoxy monomer using formaldehyde.

### 4.2 Phenolic coatings

Cardanol can also be utilised as an alternative monomer in phenol-formaldehyde resins. The phenol can be fully or partially replaced with cardanol, which reduces the brittleness and improves the flexibility of the resin.^145,146^ However, cardanol has a negative impact on the thermal stability of the resin, so full replacement of phenol is less desirable.

**Fig. 26:**
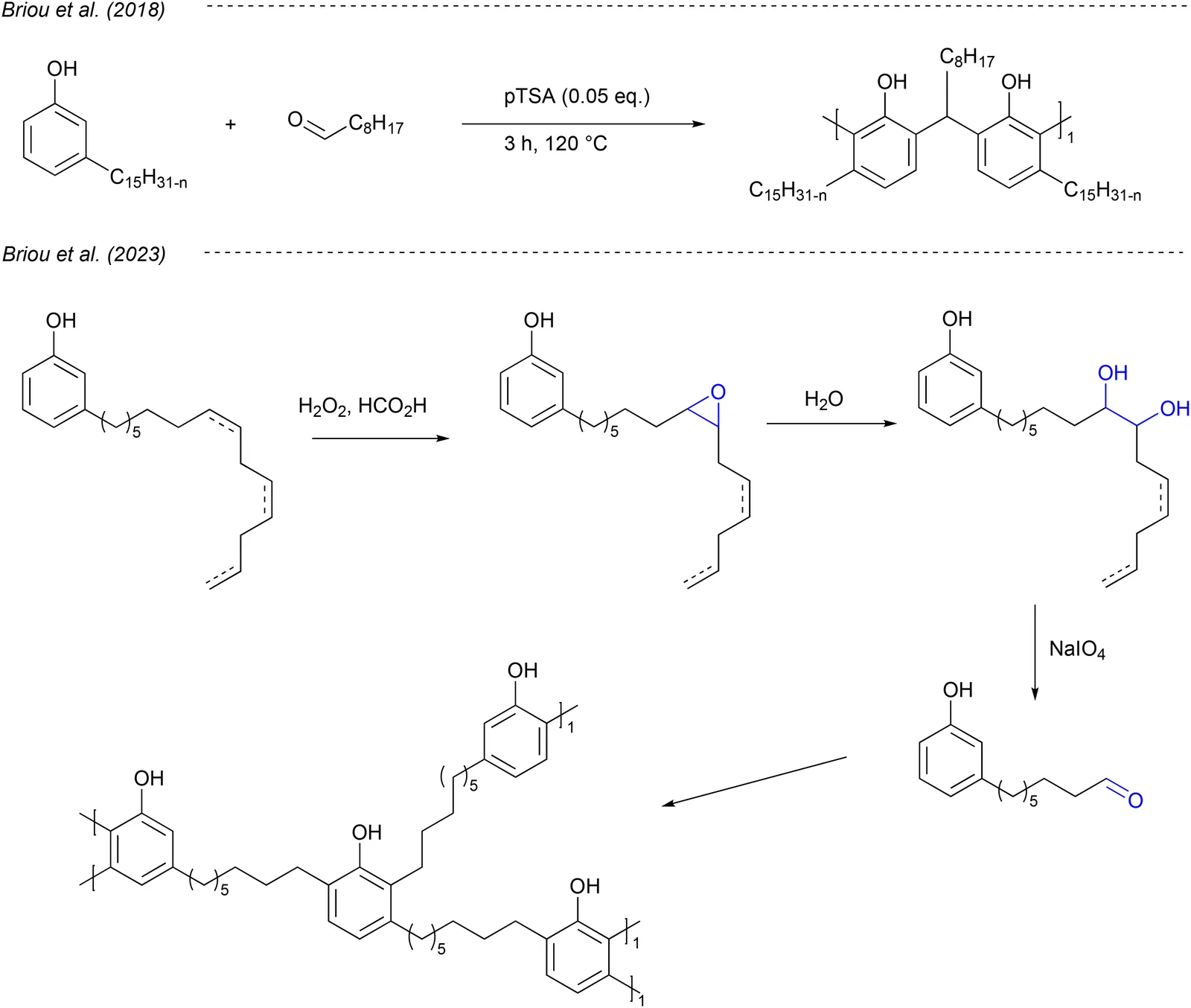
Phenolic cardanol-based coatings.

Further work in the area has looked at substituting the formaldehyde for other safer aldehydes, including the work of Briou *et al.* who utilised nonanal, which can be obtained through the ozonolysis of vegetable oil, as the aldehyde reacting with cardanol to form a flexible phenolic resin (Fig. 26).^147^ The resultant bio-based resin had a low *T*_g_ and good thermal stability.

Briou *et al.* have also developed a new method for synthesising phenolic resins, which only uses cardanol, by chemically modifying it to form 8-(3-hydroxyphenyl)octanal (Fig. 26).^148^ This involves a 3-step synthesis whereby cardanol is first epoxidised, then hydrolysed to form diols, and finally oxidatively cleaved using NaIO_4_. The aldehyde produced can then be used as an AB monomer, to form a thermally stable and low *T*_g_ phenolic resin through step growth homopolymerisation.

### 4.3 Polyurethane coatings

Cardanol has been utilised in PU resins to improve their thermal stability.^149^ As such, there have been multiple methods developed to derivatise cardanol into polyols suitable for PU resin synthesis.

**Fig. 27:**
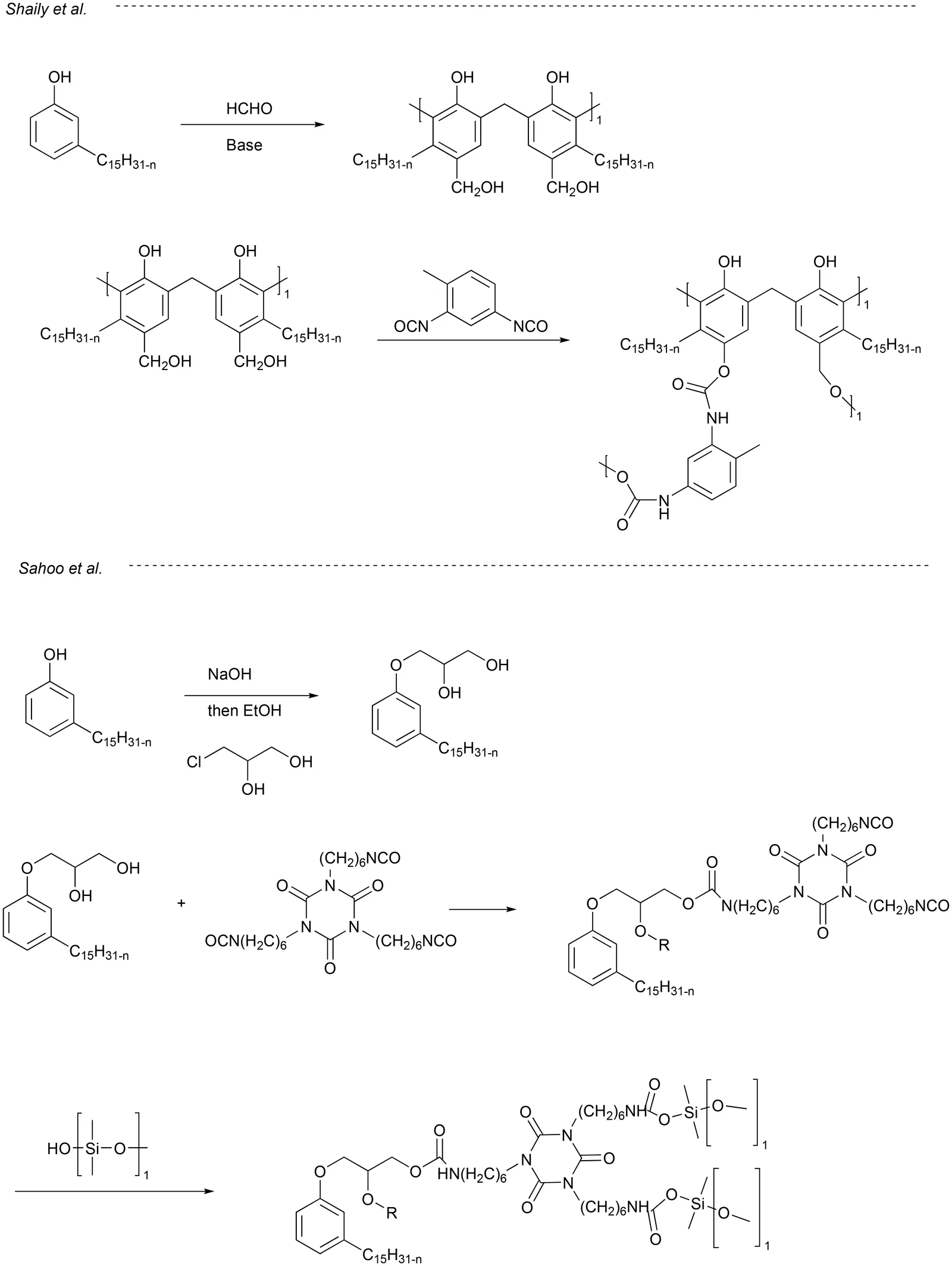
PU cardanol based coatings.

Shaily *et al.* first formed a cardanol polyol by reacting cardanol with formaldehyde, subsequently producing a PU resin by reaction with toluene-2,4-diisocyanate (Fig. 27).^150^ The effect of temperature was also tested by curing the films at 80 °C and at room temperature and it was found that the resins cured at 80 °C had better chemical stability and hydrophobicity than those cured at room temperature. The resins had good mechanical and thermal stability and showed potential for use in anti-corrosive materials.

Sahoo *et al.* derived cardanol 2,3-dihydroxy propyl ether (CPE) from cardanol, through an S_N_2 reaction with 3-chloro-1,2-propanediol. The CPE was then reacted with an aliphatic isocyanate to form the PU resin (Fig. 27).^151^ Hydroxy-terminated polydimethylsiloxane was added in varying amounts to form co-polymers with improved hydrophobicity and anti-corrosive properties. The coatings produced were colourless and transparent, whilst most previous cardanol-based PU resins were opaque and dark brown.

**Fig. 28:**
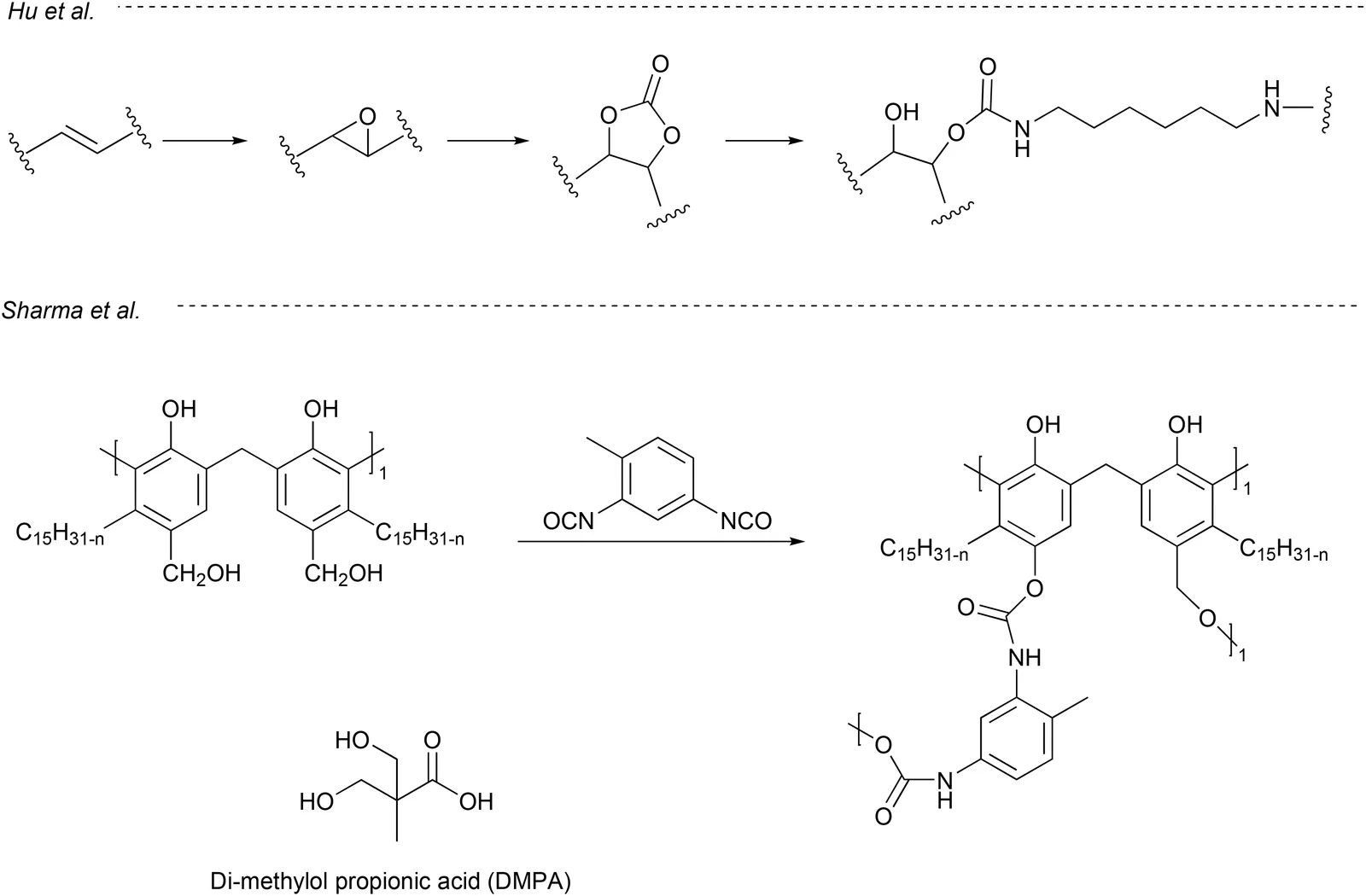
PU cardanol based coatings.

Hu *et al.* produced NIPU resins from cardanol, by utilising CO_2_. Cardanol was first epoxidised, then reacted with CO_2_ to form cyclic carbonates. These cyclic carbonates underwent ring opening using 1,6-hexanediamine to form the PU resin (Fig. 28).^152^ To improve flame retardancy, the synthesis was modified to include the use of the phosphorus species, PDP. This produced a PU resin with good flame retardancy, as well as improved thermal stability and mechanical properties. The use of not only cardanol as a bio-based source for the resin, but also CO_2_, which could hypothetically be accessed from flue gases on scale, gives a resin with a high bio-based content. The crosslinker for the resin, 1,6-hexadiamine is currently produced from petrochemicals, however future work could explore the use of bio-based crosslinkers to produce fully, or almost fully, bio-based resins.

WPUs that are hydrophobic, high solid and nano-dispersed in solution can also be derived from cardanol. Sharma *et al.* utilised methyolated cardanol and toluene-2,4-diisocyanate as the basis for the WPU resin (Fig. 28).^153^ DMPA was added as an internal emulsifier, along with methyl ethyl ketone which was used to control viscosity. This produced coatings with excellent physico-mechanical properties, as well as good hydrophobicity and chemical stability.

## 5. Other xylochemicals

A range of other xylochemicals have found use in coating applications. Lignin, itself one of the components of lignocellulose, has been utilised as an alternative to phenol-formaldehyde adhesive resins due to its highly aromatic structure.^154^ Recent advances include a one-pot strategy to develop lignin-based adhesives, making use of alkaline deep eutectic solvents (ADES).^155^ The blending of triethyl benzyl ammonium chloride and ethanolamine produced an ADES that, when heated with formaldehyde and lignin, instigated one-pot depolymerisation, demethoxylation and hydroxymethylation of lignin. This enabled cross-linking *via* formaldehyde condensation during hot-pressing to form an adhesive coating. The lignin-based adhesive demonstrated excellent strength, and formaldehyde emissions (0.076 mg mL^−1^) were significantly below regulatory standards.

Lignin has also found applications in antiviral coatings for metal surfaces.^156^ Many antiviral coatings involve metal-based materials or synthetic polymers, which can lead to difficulties in large-scale manufacturing. Antiviral coatings from five types of lignin were prepared, with soda lignin and phenolated terephthalic aldehyde-protected lignin demonstrating ability to inactivate the herpes simplex virus type 2. This was attributed to the generation of reactive oxygen species *via* the oxidation of aromatic hydroxyl groups, meaning the antiviral surface is not consumed and does not need to be regenerated to maintain the antiviral properties. Over six months, no loss in antiviral activity was observed.^156^

Lignin has been utilised directly for anti-corrosive coatings with a range of potential applications.^157^ Various groups have utilised the modification of lignin with epoxides or carbonyls and subsequent cross-linking with diisocyanates to produce anti-corrosive PU coatings.^158,159^ Waterborne epoxy metal coatings with excellent anti-corrosive properties have been developed from the modification of lignin with quaternary ammonium salts,^160^ and epoxy coatings for carbon steel have also been developed that incorporate acetylated lignin, DGEBA and isophorone diamine.^161^ The addition of acetylated lignin was shown to improve corrosion resistance by improving barrier properties, preventing physical corrosion.

The incorporation of sugars into coatings has been investigated, such as the methacrylate resins derived from xylofuranose and galactarate synthesised by Voit *et al.*^162^ By comparison with a commercial petrochemical resin urethane methacrylate 121, the galactarate and xylofuranose resins were found to have curing temperatures 20–50 °C lower, with comparable mechanical properties. These bio-based thermoset resins demonstrated high thermal resistance, as well as resistance to chemical and enzymatic degradation. The diglycidyl ether of isosorbide, a derivative of glucose, has been utilised in bio-based epoxy-silica resins.^163^ The diglycidyl ether was cross-linked with 4,4′-methylenebis(cyclohexylamine), a cycloaliphatic diamine, to produce a thermally stable cross-linked network. The addition of alkylalkoxysilanes was found to improve mechanical properties for the desired stone preservation applications.

Monoterpenes, such as limonene, have been investigated as bio-based alternatives to BPA for resins for 3D printing and photocurable thermoset applications.^164^ Recent advances have expanded upon this work to produce a range of acrylate resins derived from limonene and nopol.^165^ Terpenes have high boiling points and are not volatile. Therefore, when the UV-curable coatings were applied, these resins demonstrated a 98% reduction in VOC emissions after 2 hours compared to styrene resins. The terpene resins contained up to 75% bio-based content and showed high hardness and solvent resistance. The differing terpenes led to a range of glass transition temperatures, showing the potential for the tunability of these resins depending upon the desired coating applications.

Terpenes have demonstrated anti-fungal properties and thus have been investigated for anti-fungal wood coatings.^166^ Synthesised from commercial isobornyl methacrylate (IBOMA) and tetrahydrogeraniol acrylate (THGA), these resins showed an improved fungal stain resistance and equivalent durability to existing petrochemical coatings.^167^ THGA coatings demonstrated higher anti-fungal activity than IBOMA coatings, but were more prone to UV-degradation, hence IBOMA-based coatings may be more suitable for outdoor applications. Further study is required to assess the long-term viability of these coatings, particularly with respect to moisture resistance.

Other bio-based aromatic molecules that have been utilised in coatings include, eugenol (modified with mercaptoethanol) which has been utilised as a reactive diluent in photocurable coatings as a potential alternative to styrene-based diluents.^168^ Resorcinol-formaldehyde resins have been developed that demonstrate anti-corrosive and anti-fouling properties.^169^ Benzoxazine coatings derived from guaiacol have shown anti-corrosive potential.^170^ Protocatechualdehyde has been utilised in flame retardant wood coatings.^171^ Sustainable methods of producing lacquer paints from eco-friendly pigments and waterborne catechol resins with good surface adhesion have been developed.^172^ Lignin-derived guaiacol has been used to design microporous polymers that could be used as hydrophobic, UV-blocking coatings.^173^ This selection shows the vast potential for utilising the library of xylochemicals for a diversity of coating applications.

Summary tables of the materials discussed in this review are provided below (Tables 1–3).

**Table 1:** Summary list of referenced furfural- and HMF-derived materials, showing comparison of evaluated properties and performance

Material	Author	Commercial standard/comparison	Properties evaluated	Intended application	Performance
Phenol-furfural-clay resin	Rivero	Phenol formaldehyde resin	Thermal degradation, fire resistance	Aluminium coatings (automotive and electrical applications)	Fast rate of combustion, shorter duration of flame
Cresol-furfural-formaldehyde resin	Depta	Phenol formaldehyde resin	Physical and mechanical properties	Film forming agents in laquer, impregnating resin for laminate	Etherified resins showed comparable flexibility to etherified phenol-formaldehyde resins, improved hardness
Poly(furfuryl alcohol) resin (and long oil modified)	Seyedlar	Polyimides	Thermal stability, adhesion	Resins for heat resistant coating	Good flexibility and adhesion, worse thermal stability when modified with long oil
Furanic esters (*coating additive*)	Dong	Copper-based additives in marine coatings	Antibacterial, algal inhibitory, and biological activity properties (and coating physical properties)	Anti-fouling coatings	Lower coating hardness (meets service requirements), significant anti-microbial and anti-algal activity, low toxicity
UV-cure thiol–ene thermosets	Pezzana	Petrochemical thermosets	Thermo-mechanical properties	Functionalisable films	Comparable performance to existing bio-based thiol–ene thermosets
Furfurylamine-itaconic acid epoxy (polyester)	Pezzana	DGEBA epoxy	Thermo-mechanical properties, surface hydrophobicity	Functionalisable films, steel coatings, construction adhesive	Room temperature curing, improved *T*_g_ over existing bio-based epoxy resins
Phosphorylated furanic amine	Chen	Triphenyl phosphate	Thermal and flame retardant properties	Flame retardant for cotton and PLA	Improved self-extinguishing properties over PLA
Furanic epoxy thermoset	Tang	DGEBA-epoxy	Thermal stability, mechanical strength and flame-retardant properties	Bio-based & flame retardant epoxy	Effective flame retardancy (meets service requirements)
Phosphorylated furanic amine (*coating additive*)	Tang	Triphenyl phosphate	Flame-retardant properties	Bio-based & flame retardant additive for coatings	
Furanic diol glycidyl ether coatings	Shafranska	DGEBA epoxy	Thermal stability, hardness, flexibility, adhesion.	Steel coatings (automotive), construction adhesive	Resins are coloured, lower thermal stability, lower *T*_g_, lower toxicity than BPA, comparable hardness and adhesion to DGEBA
FDCA epoxy resin	Deng	DGEBA and DGT epoxy	Thermal stability, mechanical properties	Coatings, adhesives	Higher curing reactivity and *T*_g_, comparable mechanical properties and thermal stability
FDCA polyurethane coating	García González	Fossil-fuel-based PU coatings	Full LCA, adhesive properties, wettability, hardness, thermal stability	Adhesives, paints, coatings	Up to 79% reduction in GHG emissions, better adhesion, more moisture-sensitive
WPU dispersions	Papadopoulos	Isophthalic acid-derived PU	Thermo-mechanical properties	Waterborne coatings	Improved thermal and mechanical properties, enhanced dispersions and films
Furan-maleimide epoxy coatings	Fang	DGEBA epoxy	Self-healing properties, hardness, durability	Self-healing coatings	Furan-maleimide interactions improved self-healing, slight reduction in mechanical performance, increased yellowing
Furan-maleimide polyacrylate coatings	Fortunato	Polymethylmethacrylate	Self-healing properties, optical performance	Self-healing optical coatings, luminescent solar concentrator	Improved self-healing, comparable optical performance
Furan-maleimide cross-linked PU composite	Xie	Poly(3,4-ethylenedioxythiophene) PU coating	Self-healing properties, conductivity	Conductive self-healing coatings	Induced self-healing, reduced conductivity
Furan-maleimide epoxy coatings	Dong	DGEBA epoxy	Self-healing, adhesion, optical properties	Wood and glass coatings	Low molecular weights, lower adhesion in resins with greater self-healing
Furan-ferulic acid epoxy coating	Ye	DGEBA epoxy	Self-healing, thermo-mechanical properties	Thermoset coatings	Induced self-healing, improved *T*_g_ and tensile strength

**Table 2:** Summary list of vanillin-derived materials, showing comparison of evaluated properties and performance

Material	Author	Commercial standard/comparison	Properties evaluated	Intended application	Performance
Schiff base covalent adaptable networks (CANs) based on tris(4-formyl-2-methoxyphenyl) Phosphate (TFMP)	Wang	General petroleum based	Flame-retardant, high-performance properties	Bio-based thermosetting materials	High *T*_g_, tensile strength and flame-retardancy (high LOI, UL-94 V0 rating and V1)
					High malleability of Schiff base (bond exchange of 49–81 kJ mol^−1^)
					Rapid reprocessing with good monomer recovery
Melamine-vanillin-phosphorus (MEVAP) based PU	Borse	Commercial petroleum-based polyols and PU-prepolymers	Thermal and flame-retardant properties	Flame retardant coating for mild steel panels	Increased char yield, degradation temperature, thermal stability and bio-based content
					Increased limiting oxygen index (LOI)
					Self-extinguishing behaviour
Ethylenediamine-vanillin-phosphorus (VEDAP) based PU	Mestry	Commercial petroleum-based polyols and PU-prepolymers	Thermal and flame-retardant properties	Flame retardant coating for mild steel panels	Increased char yield, degradation temperature, thermal stability and bio-based content
					Increased limiting oxygen index (LOI)
					Self-extinguishing behaviour
Vanillin-Based Flame-Retardant Polyurethane (FRBPU) from vanillin-based diol (VDP)	Yang	Compared to BPU (polycaprolactone diol PCL + hexamethylene diisocyanate HDI)	Mechanical strength, toughness, crystallinity, flame-retardant and reprocessing properties	Not specified. Mechanically strong, flame-retardant coatings	Increased glass transition temperature (*T*_g_)
					Increase tensile strength and elongation at break
					Increased flame-retardancy (increased LOI)
					Decrease of crystallinity
Bio-based polyurethane networks (NVP) from vanillin-based diol containing organic phosphorus moieties (VD)	Hu	Compared to NVP-0 (isophorone diisocyanate IPDI + polytetramethylene ether glycol PTMG + naringenin)	Flame retardant, self-healing, antioxidative, reprocessing properties	Flame retardant coatings, wound dressing membranes, fire-control engineering materials	Increased thermal stability, mechanical strength, *T*_g_, and flame retardancy (increased LOI)
					Good self-healing and antioxidative properties (radical scavenging abilities)
WPU-PHAM *i.e.* castor-oil-based waterborne polyurethane (WPU) with vanillin-derived monomer (PHAM)	Li	Compared to WPU (castor oil + IPDI + DMBA)	Self-healing, anticorrosive, reprocessing and mechanical properties	Not specified. Mechanically strong and anticorrosive coatings	Increased tensile strength and decrease of elongation at break
					Increase self-healing and remodelling properties
					Chemical degradation behaviour
					Anticorrosive properties upon GO nanoparticles addition
WPU incorporating a vanillin-derived diol chain extender (WPU-VAN-OH)	Banan	Compared to WPU (IPDI + PTHF + DMPA)	Mechanical and self-healing properties	Designed for protective coatings, advanced adhesives, flexible elastomers, stainless steel coatings	Increased tensile strength
					Increased thermal stability
					Decreased water absorption
					Great adhesion and self-healing properties
VASB-WPU *i.e.* waterborne polyurethane (WPU) based on a vanillin-derived aromatic Schiff base (VASB)	Chen	Compared to WPU (IPDI + PTHF + DMPA + MDEA + PETA)	Mechanical and self-healing properties	Self-healing wood coatings	High healing efficiency upon stimulation maintaining mechanical performance
					High self-healing properties
					High toughness and tensile strength and shape memory capabilities
CWPU-VSD *i.e.* castor oil-based waterborne polyurethane (CWPU) with vanillin-based diol (VSD)	Zhou	Compared to CWPU (castor oil + IPDI + DMBA)	Mechanical and self-healing properties.	Paper-based barrier coatings	Fine-tunning of tensile strength
					Self-healing properties, hydrophobicity, low water vapor permeability
					Good recyclability and degradability.
Divanillin (DV) for PUs	Mahajan	Compared to PU (PEG + IPDI + DMPA + HEMA)	Mechanical properties	Wood coatings	Increased pencil and scratch hardness
					Increased acid and moderate alkali resistance
VBD-TMP_*x*_*i.e.* vanillin-based diol (VBD) with l-lysine diisocyanate (LDI) and trimethylolpropane (TMP)	Zhao	Commercial thermosetting PUs and petroleum-based PUs	Mechanical properties and degradability	Various coatings. Matrix material for fibre-reinforced plastics, optical materials	High toughness and comparable mechanical properties to commercial thermosetting PUs
					Complete degradability and high bio-based content
					Transparency
FDV-OH and TPV-OH based PUs *i.e.* polyols from FDCA-vanillin (FDV–OH) and TPA-vanillin (TPV–OH) with MDI methylene diphenyl diisocyanate (MDI) and hexamethylene diisocyanate (HDI)	Luo	Petroleum-based PUs	Mechanical properties and degradability	Not specified. Coatings and packaging	High mechanical strength, thermal stability and solvent resistance
					Tuneable *T*_g_
					High degradability and bio-based content
Vanillin-based poly(urethane urea) networks (VPUs)	Wang	Petroleum-based PUs	Ice-phobic and self-healing properties	Anti-icing outdoor coatings at extreme cold, acidic and alkaline conditions	Low ice adhesion
					High chemical stability
					Anti-fouling and high self-healing properties
					High lifespan with properties maintained after recycling
NIPU oligomers	Gérard	Petroleum-based PUs	Synthesis of the material evaluated	Not specified	Properties of the coating not evaluated
NIPHU CAN	Nuñez Tapia	Petroleum-based PUs	Thermal, mechanical, self-healing properties and recyclability	Not specified coatings	Favourable thermal and mechanical properties
					Increased bio-based content
					Self-healing properties
					Recyclability under mild conditions
DGEVA epoxy resin	Petrusonyte	DGEBA epoxy resin	Rheological, mechanical and thermal properties	Not specified	High storage modulus, loss modulus, complex viscosity, *T*_g_ and Young's modulus
DGEVA-silica epoxy coating	Trentin	DGEBA epoxy resin	Anti-corrosive and adhesion properties.	Not specified	High adhesion and thermal stability
					Anti-corrosive properties
					High lifespan
DGEVA epoxy resin reinforced with nanoclay	Noè	DGEBA epoxy resin	Anti-corrosive properties	Not specified	High anti-corrosive properties
					Comparable surface tension and hardness to petroleum-based epoxies
PTEP-OSL/ZnO coating	Wei	DGEBA epoxy resin	Anti-corrosive and hydrophobic properties	Not specified/glass coatings	Super-hydrophobicity
					Good anti-corrosive properties
					High bio-based content
					Smooth surface
					High impedance
VDGE-GDMA epoxy resin	Motiekaityte	DGEBA epoxy resin	Anti-microbial, mechanical and thermal properties.	Anti-microbial coatings, films and optical 3D printed objects	Higher mechanical and thermal properties with GDMA increase and higher anti-microbial properties with VDGE increase
HO-SBM and GVA copolymers	Dominich	DGEBA epoxy resin	Mechanical properties and dual-curing capabilities	Not specified/mild steel panel coatings	Increased chemical resistance, hardness and Young's modulus depending on copolymer composition
SA-ODA-EP/DDM	Cai	DGEBA epoxy resin	Anti-microbial, flame retardant and mechanical properties	Not specified	Flame retardant properties (high LOI)
					Great mechanical properties (high *T*_g_ and storage modulus)
VEV coatings	Wang	DGEBA epoxy resin	Superhydrophobic, anti/deicing and self-healing properties	Not specified/superhydrophobic coatings	High anti-icing performance
					High photothermal deicing performance
					Self-healing, recycling and hydrophobic properties
					High bio-based content
APGO-based epoxy vinyl ester resins	Xu	Petroleum-based epoxy vinyl ester resins	Anti-corrosive properties	Metal coatings in marine environment	High mechanical strength and thermal stability
					Low conductivity
					High anti-corrosion efficiency
					High impedance modulus.
EP/MXene@ZIF	Xue	Petroleum-based epoxy resins	Anti-corrosive, mechanical and flame-retardant properties.	Not specified	Reduced heat and smoke release
					High flame-retardant properties (high LOI)
					Good long-term anti-corrosive properties
					High impedance and enhanced mechanical properties
Acetal-based epoxy monomer CA4 cured with different diamines	Li	Polymer networks derived from acetal-based epoxy monomers to enhance recycling properties.	Mechanical properties and degradability.	Carbon fibre reinforced polymer (CFRP) composites and recyclable thermosets.	High tensile strength
					Fast degradation under mild conditions.
					Carbon fibre recycling
					High Young's modulus and thermal stability
Tricyclic acetal curing agent (TVS) for degradable epoxy resins.	Song	Petroleum-based epoxy resins. Improving epoxy networks with dynamic acetal bonds for recycling properties.	Modulus, thermal stability and degradability.	High-performance polymer-based composites.	High tensile strength and thermal stability
					Fast degradation under mild acidic conditions
					Upcycles into carbon fibre composites.
Acetal epoxy 1, 2 and 3 (AEp-1, AEp-2 and AEp-3) cured with IPDA	Li	Addressing processability of degradable acetal-based epoxy thermosets.	Viscosity, thermal and mechanical properties, degradability.	Degradable epoxy monomers with low viscosity for degradable thermosets.	Low viscosity
					Favourable mechanical and thermal properties
					Controllable degradability
					Used to prepare carbon fibre composites with high recovery of carbon fibres upon epoxy matrix degradation
VAEI epoxy curing agent	Xu	Petroleum-based epoxy resins	Anti-microbial, anti-corrosive and low dielectric properties	Coatings for electronic devices	High impedance modulus
					Low dielectric constant
					Good anti-microbial properties
OV-DDM/APP epoxy curing agent	Wang	Hexahydro bisphenol A diglycidyl ether (DEMBA)	Flame-retardant and mechanical properties.	Not specified	Values dependant on the final composition of curing agent and epoxy resin
					High LOI and flame-retardant index (FRI)
					High thermal stability
					Small increase in storage modulus and mechanical properties
VB/DDM epoxy curing agent	Li	DGEBA epoxy resin	Flame retardant properties	Wood coatings	Improved thermal stability and flame retardancy
					High LOI
					Lowered smoke and heat emission
VAPD-5/EP coating	Liang	DGEBA epoxy resin	Self-healing, flame-retardant, UV-resistant properties.	Wood coatings	Complete self-healing
					UV-shielding properties
					High LOI, hardness and adhesion
E51/VAN-D230 and GOP coating	Yang	DGEBA epoxy resin	Mechanical and anticorrosive properties	Marine environment coatings	High tensile strength, bending strength, storage modulus at room temperature, and *T*_g_
					Excellent stress relaxation properties, ageing resistance and anti-corrosive properties
PPV	Xanthopoulou	Poly(ethylene terephthalate) (PET), poly(butylene terephthalate) (PBT)	Thermal attributes and crystallisation profile.	Not specified. Not designed as a coating	
PHV	Xanthopoulou	Poly(ethylene terephthalate) (PET), poly(butylene terephthalate) (PBT)	Thermal and mechanical properties, and crystallisation and recyclability	Not specified. Not designed as a coating	Great elastic recovery behaviour
					Low processing temperature
					High recyclability.
PBT-VANI	Dong	Poly(butylene terephthalate) (PBT)	Adhesive properties	Hot-melt adhesives	Lower melting temperature
					High adhesion strength
					Great single-lap shear strength
					Thermal and chemical resistance
					High recyclability
VMA-*x* copolymers	Liu	Petroleum-based acrylates	Anti-fouling properties	Marine coatings	Anti-microbial properties
					Environmentally friendly anti-fouling coating
PRE coatings	Liu	Petroleum-based acrylates	Anti-fouling properties	Marine coatings	Anti-bacterial and anti-algae activityGood adhesion
					Low surface energy
					Resistance to prolonged immersion in water
D-20P and B-20P	Kazlauskaite	Petroleum-based acrylates	Anti-microbial properties	Antimicrobial films, coatings, and 3D printed parts. Materials suitable in medicine, electronics, and robotics	Self-healing and self-welding properties
					Increased mechanical properties
					Recyclability
BIO-X (containing VDDM)	Ozukanar	Petroleum-based acrylates	General properties	Metal coating. Photocurable coil coatings.	High bio-based content
					Good metal adhesion
					Increased *T*_g_
					High hydrophilicity
					Anti-corrosive properties
					Recyclability
Vanillin MMA	Jašek	Petroleum-based acrylates	UV-shielding	Wood coatings	High tensile modulus and tensile strength
					High adhesive and UV-shielding properties
Poly(VA-xxx)	Appasamy	Thermosetting phenolic resins	Hydrophobic and anti-corrosive properties	Water repellent and anti-corrosive coatings. Mild steel coatings.	High thermal stability
					High heat resistance index
					High char yield and hydrophobicity
					Anti-corrosive properties
Poly(VG-amine) and Poly(VG-sa)/BS	Mohanraj	Traditional petroleum-based polybenzoxazines with petroleum-based phenols	Thermal and anti-corrosive properties	Not specified	Depending on the amine used: improved thermal resistance, water repellence, and anti-corrosive properties, improved hydrophobicity, high corrosion protection
PBZ-Silica hybrid composites	Perumal	Traditional petroleum-based polybenzoxazines with petroleum-based phenols	Oil–water separation applications and anticorrosive properties	Surface modified cotton fabric, mild steel panel coatings	Depending on the composition: high oil–water separation efficiency and water flux value, high impedance and anti-corrosive properties, anti-icing properties
Vanillin-based polybenzoxazine-silica (PBZ-Si) hybrid nanocomposites	Perumal	Traditional petroleum-based polybenzoxazines with petroleum-based phenols.	Oil–water separation applications and anticorrosive properties	Cellulose and mild steel coatings	Enhanced thermal stability
					Good oil–water/oil–water emulsion separation
					Anti-corrosion properties
					High water contact angle and oil flux value
nCeO_2_/PBZ-co-PU nanocomposite	Meera	Traditional petroleum-based polybenzoxazines with petroleum-based phenols	Anti-corrosive properties	Mild steel panel coatings. Adhesives and anti-corrosive coatings.	High corrosion resistance
					High surface hydrophobicity

**Table 3:** Summary list of referenced cardanol-derived materials, showing comparison of evaluated properties and performance

Material	Author	Industry standard/comparison	Properties evaluated	Intended application	Performance
Cardanol epoxy	Caillol	DGEBA epoxy resin	Thermal stability, *T*_g_, gloss, hardness	Thermoset polymers/epoxy polymers	With additives, shows comparable properties to DGEBA epoxy, lower *T*_g_
Cardanol epoxy	Prasad	DGEBA epoxy resin	Thermal, mechanical, anti-corrosive properties	Steel coatings	State enhanced mechanical, thermal and anti-corrosive properties (*versus* without additive)
Phosphorous-containing cardanol curing agents	Huang	DGEBA epoxy resin	Flame retardancy, mechanical properties, shape memory	Flame-retardant epoxy	Improved flame retardancy and toughness
Phosphorous-containing cardanol curing agents	Chen	DGEBA epoxy resin	Flame retardancy, mechanical properties	Carbon nanofibre reinforced composite	Improved flame retardancy
Epoxy cardanol and cardanol-derived phosphate	Wang	DGEBA epoxy resin	Flame retardancy, thermal stability, mechanical strength	Epoxy thermosets	Improved flame retardancy, improved tensile strength/toughness over DGEBA
Cardanol-nonanal resin	Briou	Phenol-formaldehyde resin	Thermal stability, mechanical properties	Soft bio-based thermosets	Lower hardness, no loss of thermal stability
Cardanol-phenolic resin	Briou	Phenol-formaldehyde resin	*T*_g_, thermal stability	Phenolic thermosets	Low *T*_g_, good thermal stability
Cardanol PU resin	Shaily	Non-cardanol PU resin	Chemical stability, hydrophobicity, mechanical and thermal stability	Protective, anti-corrosive and decorative coatings	Good chemical and thermal stability, good mechanical strength, excellent flexibility
Cardanol PU resin	Sahoo	Non-cardanol PU resin	Hydrophobicity, anti-corrosive properties, appearance	Anti-corrosive and hydrophobic colourless transparent coatings	With addition of HTPDMS additive: improved hydrophobicity and anti-corrosive properties, good adhesion strength and thermal stability
NIPU cardanol resin	Hu	Non-cardanol PU resin	Flame retardancy, thermal stability, mechanical properties	Synthetic NIPU explored as an adhesive	Improved flame retardancy, Adhesion strength up to 2.47 MPa
WPU cardanol resin	Sharma	“traditional solvent-based coatings”	Hydrophobicity	Protection of steel surfaces	Corrosion inhibition efficiency of approximately 98.98% over 24 days in a 3.5% NaCl solution

## 6. Conclusions

Xylochemicals are being widely explored for applications in coatings as a consequence of the desire to move away from reliance on petrochemicals. A permanent intention across the three xylochemicals groups explored in this review is that, irrespective of the final material application, they are meant to increase the bio-based content of the final product. Across the groups though, additional motivations emerge. For example, furfural and HMF are frequently observed in the literature as direct replacements for petrochemical aldehydes due to their improved safety, and this is cited in much of the literature for phenol-formaldehyde resin replacement. As well as hazard reduction, phenol-furfural resins exhibit better solubility characteristics and are less brittle than phenol-formaldehyde resins. Several of the articles discussed in this review focus on functionalising standard phenol-furfural resins further to improve properties such as hardness and flame retardancy. The bio-based furans are also common substitutes for petrochemical BPA in DGEBA epoxies. Again, safer profiles are a key factor in these switches. Many of these furanic epoxies exhibit similar adhesion and hardness to petrochemical DGEBA; however, some of the trade-offs cited were lower thermal stability and colour in the final resin. As with the phenol-furfural resins, there are examples in the literature of functionalisation being undertaken to improve the performance of furanic epoxies, such as phosphorylation for flame retardancy and cross-linking for self-healing properties. Besides their incorporation as monomers, the furans have frequently been used as platform chemicals for bio-based additives. Such additives include the furanic esters used as anti-fouling additives for marine coatings, and phosphorylated furanic amines used as flame retardants in flammable/combustible coatings.

Vanillin has been employed extensively and in a variety of novel coatings in the literature, including bio-based PUs, DGEBA replacements, PET alternatives, marine coatings, and more. This is largely due to the significant functionality it possesses. The anti-microbial properties of vanillin also make it an ideal bio-based replacement in anti-fouling applications, either as an additive or incorporated as a monomer into marine coatings. The handle frequently exploited on vanillin is the aldehyde group, which is extensively used with diamines to produce Schiff base polyols. Such polyols are then deployed in prepolymers and as chain extenders for PUs. A novel property that comes about from this dynamic covalent chemistry is self-healing, which is observed in many vanillin-derived imine PUs. This offers a significant sustainable improvement over many petrochemical PUs beyond simply increasing bio-based content. The rich functionality of vanillin offers a route to circular polymers and coatings whilst providing similar physicochemical properties (aromaticity and rigidity) that benchmark petrochemical monomers like styrene and BPA offer.

Cardanol, like vanillin, is a bio-based chemical with rich functionality, but is typically employed for further functionalisation for specialised applications. In this way, it is used simultaneously as a bio-based replacement and a platform monomer to achieve novel/improved properties. A frequently observed example of this in the cardanol literature is phosphorous functionalisation for deployment in epoxy resins. This addresses bio-based content and flammability of DGEBA epoxies simultaneously. A cited drawback of cardanol for these applications is that the *T*_g_ of the epoxy is too low. Cardanol-based phenolic resins in the literature also cited low *T*_g_ in final polymers. Whilst the properties of cardanol may be suitable for specific low *T*_g_ or hydrophobic polymer applications, they pose a problem for the use of cardanol as a drop-in for existing petrochemical aromatic monomers.

A common theme noted over the course of this review is that, despite advancements, it can be difficult for these new sustainable materials to make it to market. It is not enough for these materials to be more sustainable than existing coatings; they must show improved properties and be economically viable for them to be taken to an industrial scale. One of the greatest obstacles to viable bio-based coatings is that, in many cases, the starting materials are either not produced on a large scale from biomass or are produced on a larger, more economical scale from petroleum sources.

Rigorous comparison against a standardised set of performance criteria, processing requirements, economic feasibility, sustainability benefits and remaining technical challenges is not possible from early Technology Readiness Level (TRL 1–3) research shared in the literature. A more cohesive approach is required if the disparate research described in this review is to stand the best chance of yielding viable challengers to the incumbent polymers.

The petrochemical processes have typically undergone many years of optimisation and investment inevitably making the petrochemical cheaper than the bio-based equivalent. In order to facilitate industrial adoption of these materials, not only is investment needed in the research of the materials, but legislative pressure and a joint effort of academia and industry is needed to demonstrate their viability.

The following recommendations are made for both near and longer term research into incorporation of xylochemicals for paints and coatings.

Near term: fundamental property characterisation, including, but not limited to: *T*_g_, impact resistance, photostability, corrosion resistance, healing efficiency for self-healing coatings, and structure property relationships, to permit benchmarking against typical incumbent systems. Longer term: robust life cycle assessment (LCA) of the most promising candidates, comparing bio-based coating systems with petrochemical analogues across a range of functional units, explicitly addressing concerns around agricultural land use, co-product allocation, and end-of-life scenarios, including approaches facilitating the targeted separation of coatings and underlying substrate to facilitate recycling.

The efforts to implement a circular economy are ongoing, but it is hoped that with further research and development, the implementation of more sustainable materials will make this an achievable reality.

## Author contributions

M. J. D., J. M. G and M. S. S wrote the original draft, H. M. helped with writing – reviewing and editing, H. F. S conceived the review and helped with writing – reviewing and editing.

## Conflicts of interest

There are no conflicts to declare.

## Data Availability

No primary research results, software or code have been included and no new data were generated or analysed as part of this review.
